# Encoding-related hippocampus connectivity for scenes, faces, and words: Healthy people compared to people with temporal and frontal lobe epilepsy

**DOI:** 10.1016/j.nicl.2025.103784

**Published:** 2025-04-12

**Authors:** Anna Doll, Daniel A. Schlueter, Martin Wegrzyn, Friedrich G. Woermann, Kirsten Labudda, Christian G. Bien, Johanna Kissler

**Affiliations:** a Bielefeld University, Medical School and University Medical Center OWL, Mara Hospital of the Bethel Foundation, Department of Epileptology, Bielefeld, Germany; b Bielefeld University, Department of Psychology, Bielefeld, Germany; c University Hospital OWL, Bielefeld University, Evangelisches Klinikum Bethel, Department of Psychiatry and Psychotherapy, Bielefeld, Germany; d Bielefeld University, Center for Cognitive Interaction Technology (CITEC), Bielefeld, Germany

**Keywords:** Functional connectivity, Hippocampus, Frontal lobe epilepsy, Temporal lobe epilepsy, fMRI, Memory

## Abstract

Interactions of the hippocampus with other brain structures are supposed to support memory formation but knowledge is limited regarding hippocampal task-based functional connectivity (FC) during encoding in both healthy people and people with epilepsy, who frequently have impaired memory. We compared absolute [FC(encoding)] and relative FC (isolating task-specific FC [FC(encoding)-FC(baseline)]) of the anterior hippocampus in 30 controls, 56 mesial temporal (mTLE, 26 right) and 24 frontal lobe epilepsy (FLE) patients using a memory fMRI-task of encoding scenes, faces and words. **In controls**, absolute hippocampus FC comprised regions typically active in memory fMRI-tasks and the default mode network (DMN): For faces and scenes, FC was pronounced to temporo-occipital areas, whereas for words it extended to lateral-temporal regions. Relative FC was more circumscribed and encompassed temporo-occipital and frontal stimulus-selective regions for scenes and faces. Also, relative FC revealed weaker hippocampus – DMN connectivity during encoding. **mTLE** patients had decreased FC from the epileptogenic hippocampus and slight disruptions from the contralateral hippocampus. Decreased absolute FC was found to the contralateral mTL, the precuneus and the posterior cingulate gyrus. Further, mTLE patients’ weaker FC to frontal and temporo-occipital regions reflected material-specific changes. Conversely, mTLE patients had higher absolute FC to regions to which the hippocampus is normally anticorrelated and increased relative FC to DMN regions. During word encoding only, **FLE** patients had increased left hippocampal relative FC to right-sided regions. Together, these findings further delineate the network architecture of memory in healthy people and its dysfunction in focal epilepsies, which prospectively could inform surgical interventions.

## Highlights

• Task-based hippocampus functional connectivity (FC) revealed intrinsic and stimulus-specific FC in controls.
• TLE patients had widespread FC disruptions from the epileptogenic hippocampus.
• TLE patients also had slight FC disruptions from the contralateral hippocampus.
• FLE patients had less hippocampus FC disruptions occurring primarily during word encoding.

## Graphical abstract

**Figure:**
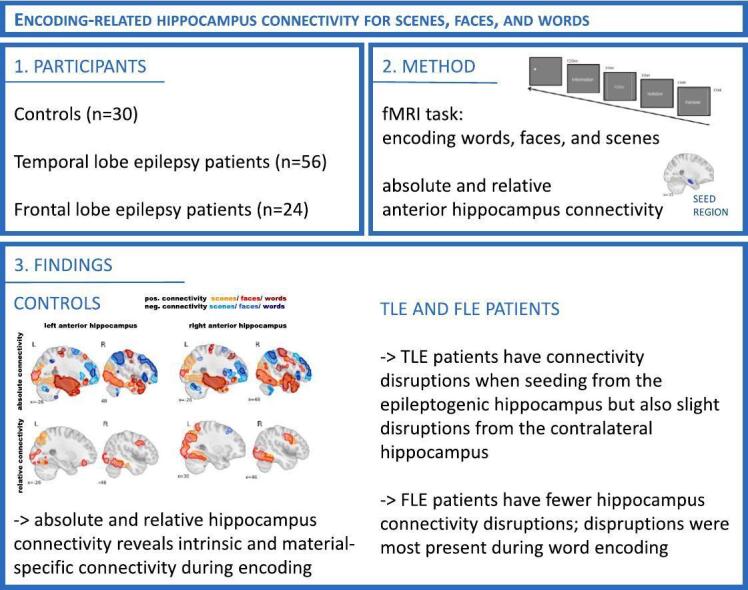

## 1 Introduction

The hippocampus is known as a key structure for successful memory formation whose dysfunction results in memory disorders, particularly when forming new memories. However, research has also shown that in order to orchestrate successful encoding, an interplay of distributed brain structures beyond the hippocampus is necessary (Jeong et al., 2015). Functional connectivity (FC) measures can be used to delineate this interplay during encoding tasks in healthy people and reveal its alterations in people with memory deficits.

In general, task-based FC can be calculated during an encoding condition. Then FC includes both task-related and remaining intrinsic FC and results in so-called “task-state FC” or “absolute FC” (*abs*-FC). Alternatively, FC can be calculated to include only task-related FC and to exclude intrinsic FC by means of contrasting the encoding condition with the baseline resulting in so-called “task-modulated FC” or “relative FC” (*rel*-FC).

Regarding task-based hippocampus FC during encoding, there is little previous research, even in healthy people. Encoding-related *abs*-FC was investigated by McCormick et al. (McCormick et al., 2010) and Foster et al. (Foster et al., 2016) for faces and words, respectively. There was overlapping connectivity in both studies, namely within and between the hippocampi, and to the parahippocampus, insula, precuneus, inferior parietal cortex and fusiform gyrus. The studies differed in that in the study by McCormick et al. the hippocampus further coactivated with frontal and retrosplenial cortices (McCormick et al., 2010), whilst Foster et al. found coactivation with temporal gyri and the posterior cingulate gyrus (Foster et al., 2016). Studies on encoding-related *rel*-FC applied paradigms with words (Fleury et al., 2022, Schott et al., 2013), faces (Fleury et al., 2022) and face-place pairs (Beason-Held et al., 2021). *Rel*-FC patterns were more circumscribed than *abs*-FC and comprised no or only little within and between mTLs connectivity. In detail, for words, Schott et al. found hippocampus *rel*-FC to be most pronounced in frontal, parietal and occipital regions with some within and between mTL connectivity (Schott et al., 2013), whilst Fleury et al. reported only connectivity to mesial and lateral temporal regions and hardly any to the frontal lobe (Fleury et al., 2022). Connectivity patterns for faces predominantly encompassed mesial and lateral temporal and frontal regions (Fleury et al., 2022). For face-place pairs, Beason-Held et al. described hippocampus connectivity to frontal, lateral temporal, and occipital regions. However, they applied three different hippocampal seeds (tail, body, head), each resulting in varying connectivity patterns (Beason-Held et al., 2021). So far, overall comparability between studies is low as seed regions, FC calculation methods and the stimuli employed varied.

Together, it remains unclear if studies on encoding-related *abs*- and *rel*-FC differ due to differences in paradigm, sample characteristics, seed selection or analysis methods or due to different stimulus materials, which would imply material-specific encoding networks. Investigating *abs*- and *rel*-FC in one study would be informative to learn more about mechanism in material-specific encoding connectivity in healthy people.

People with temporal lobe epilepsy (TLE) and frontal lobe epilepsy (FLE) are prone to memory deficits (e.g. Bremm et al., 2019, Exner et al., 2002, Chang et al., 2019). Accordingly, they show alterations during encoding in functional MRI (fMRI) tasks. Most consistently, studies observed reduced mesial temporal lobe (mTL) activation in TLE patients and, to a lesser degree, in FLE patients, especially in those with poor memory performance (e.g. Doll et al., 2021, Centeno et al., 2012, Sidhu et al., 2013, Doll et al., 2024). Besides traditional task-based fMRI activation analysis, task-based functional connectivity (FC) analysis might expand knowledge regarding cerebral alterations during memory formation in TLE and FLE, especially given that even focal epilepsies are increasingly recognized as resulting in cerebral network disorders (Kramer and Cash, 2012).

Despite the paucity of task-based FC studies in both healthy controls and focal epilepsy patients, there is a sizeable literature on resting-state FC. Defining a seed in mTL, usually the hippocampus, most studies demonstrated less ipsilateral hippocampus FC in TLE patients than in controls to regions of the default mode network (DMN), or the lateral or mesial TL during rest (Pittau et al., 2012, Doucet et al., 2013, Pereira et al., 2010, Bettus et al., 2010, de Campos et al., 2016, McCormick et al., 2013, Whitten et al., 2021). However, others also reported increased ipsilateral mTL FC in TLE patients (Roger et al., 2020); a mixture of decreased and increased ipsilateral mTL FC (Li et al., 2015, Voets et al., 2014, Haneef et al., 2014, Li et al., 2017) or no group differences (Barron et al., 2015). Regarding FC from the contralateral mTL seed, there is less evidence. Some studies again indicate decreased FC compared to controls (Pittau et al., 2012, Doucet et al., 2013, Pereira et al., 2010), others increased FC (McCormick et al., 2013, Voets et al., 2014, Li et al., 2017) or a mixture of both (Bettus et al., 2010, Li et al., 2015, Haneef et al., 2014). Therefore, the pattern of hippocampus FC in TLE requires further clarification. Moreover, to our knowledge only one study explored mTL network changes at rest in a small group of 15 FLE patients and compared TLE and FLE patients (Bu et al., 2024). They analyzed hippocampus connectivity exclusively to regions of the DMN and found no significant difference between FLE patients and controls. TLE compared to FLE patients had reduced FC from the left hippocampus to the precuneus and medial frontal gyrus.

Inconsistencies in studies investigating mTL FC in TLE patients might originate from varying seed and target regions between studies and the fact that studies had mostly rather small sample sizes with varying sample characteristics. Importantly, these might also be explained by high variance within and between subjects during resting state. To reduce this variance, recent literature recommend analysis of FC during specific tasks (Finn et al., 2017, Rosenberg and Finn, 2022) as task-based FC data is typically less noisy and has higher within-subject correlations than resting state FC (Vanderwal et al., 2017, Gonzalez-Castillo and Bandettini, 2018).

Regarding task-based FC in epilepsy patients, there is to our knowledge only one study which explored hippocampus FC during memory encoding in TLE (Fleury et al., 2022) and none in FLE. Fleury et al. used Psycho-Physiological-Interaction (PPI) analysis contrasting FC during successful encoding of words or faces compared to baseline, thus reporting *rel*-FC. They found increased *rel*-FC between bilateral mTLs and between the mTL and temporo-occipital regions in TLE patients compared with controls (Fleury et al., 2022). Whilst *rel*-FC analysis allows to draw conclusions about circumscribed task-specific FC, it also tends to lack power and reliability and might result in spurious effects due to high collinearity of different conditions (O'Reilly et al., 2012). Therefore, *rel*-FC should be complemented by *abs*-FC analysis (Cole et al., 2014).

The aim of this study was to explore anterior hippocampus *abs*- and *rel*-FC during a memory fMRI task of encoding scenes, faces and words in healthy people and in mesial TLE (mTLE) and FLE patients. Thereby we expand knowledge regarding encoding networks for different materials in healthy people and elucidate the impact of seizure origin and encoding material on memory network configuration in the most common focal epilepsies. Here, investigating both *abs*- and *rel*-FC reveals a more detailed view on encoding networks and allows us to capture disease-specific FC changes which might impact task performance, including both material-specific encoding and the more general intrinsic FC.

## 2 Materials and methods

### 2.1 Participants

We studied 30 controls and 25 FLE and 57 mTLE patients, who were consecutively recruited from the presurgical epilepsy monitoring unit at the Mara, Bielefeld, Germany. Epilepsy origin and lateralization of patients’ epileptic onset zone were ascertained by standard epilepsy presurgical diagnostics in this experienced center (Cloppenborg et al., 2016). Requirements for controls’ participation were an age ≥ 18 and absence of known neurological and psychiatric disorders. Table 1 details the demographic and clinical characteristics of the final sample.

**Table 1:** Demographic and clinical characteristics of controls, FLE, lmTLE and rmTLE patients.

	Controls *n* = 30	FLE *n* = 24	lmTLE *n* = 30	rmTLE *n* = 26
Age in years [*M (SD)* (range)]	35.4 (13.3) (19–60)	32.5 (12.7) (18–65)	40.0 (14.0) (18–58)	37.0 (11.9) (19–61)
Sex in % [male/female]	50.0/50.0	58.3/41.7	43.3/56.6	50.0/50.0
Years of schooling [*M (SD)* (range)]	10.6 (1.6) (9–13)	10.8 (1.4) (9–13)	10.7 (2.1) (4–13)	10.6 (1.7) (9–13)
Handedness [right/left]	28/2	22/2	27/3	26/0
Language laterality^a^ [right/left/bilateral/unknown]		0/22/2/0	0/26/3/1	1/22/2/1
Laterality of epileptic focus [left/right/bilateral]		8/15/1		
Age at epilepsy onset [*M (SD)* (range)]		15.1 (14.9) (0–62)	21.0 (12.0)^c^ (3–55)	23.0 (11.8) (1–57)
Epilepsy duration [*M (SD)* (range)]		17.4 (9.2) (0–34)	17.6 (13.8)^c^ (0–53)	14.0 (9.6) (0–40)
Antiseizure medication load^b^ [*Mdn* (range)]		2.6 (1.0–5.4)	2.3 (0.3–5.3)	2.4 (0.7–5.2)
Aetiology mesio-temporal sclerosis tumors/ cavernomas cavernoma + hippocampal sclerosis encephalocele + amygdala lesion focal cortical dysplasia unspecified dysplastic lesion/ cyst unspecified lesion/ no lesion		0 1/ 2 0 0 11 4/ 1 2/ 3	23^d^ 1/ 1 1 1 0 0/ 0 2/ 1	20^e^ 4/ 0 0 1 0 0/ 0 1/ 0

*Note.* There was no significant difference between controls and FLE or lmTLE or rmTLE patients regarding age, sex, years of schooling and handedness (*p*s > 0.1).

Abbreviations: FLE, frontal lobe epilepsy; lmTLE, left mesial temporal lobe epilepsy; M, mean; Mdn, median; rmTLE, right mesial temporal lobe epilepsy; SD, standard deviation.

a Language laterality was determined by a language fMRI (Woermann et al., 2003) for all but one patient, for whom it was determined by Wada test. According to our neurologist, the assessment of two of the presumably left-lateralised patients is uncertain.

b The antiseizure medication load was estimated as the sum of the ratios (prescribed daily dose/defined daily dose) for each antiseizure medication, with the doses corresponding to those defined by the World Health Organization.

c For two patients the epilepsy onset was estimated to have been with 3 years, as records specified it to have been “in earliest childhood”.

d One with additional mesial cavernoma.

e One with additional mesial heterotopia and one with additional autoimmune-encephalitis related paraneoplastic mTLE.

One FLE patient who reported to have fallen asleep during the experiment was excluded. Two additional FLE patients were excluded from the verbal encoding condition only: one right FLE patient, because of deficient German, and one left FLE patient who reported not having seen the words clearly. Further, four mTLE patients had incomplete datasets: One right mTLE (rmTLE) patient had a seizure during the last run (completing only words and scenes). For one left mTLE (lmTLE) patient, the fMRI data of scenes and faces could not be analysed due to technical issues during the recordings. Further, two lmTLE patients discontinued the fMRI task after the second run (one completed words and faces, the other one completed words and scenes). We already published the results of the activation analysis of the described sample (Doll et al., 2021, Doll et al., 2024). Here, we excluded only one lmTLE patient in FC analysis because of high motion (Morfini et al., 2023) and added one rmTLE patient based on new diagnostic information.

All participants had normal or corrected-to-normal vision. Before participation, all gave written informed consent according to the Declaration of Helsinki. The ethics committees of Bielefeld University (EUB 2017–080) and Westphalia-Lippe Medical Association (2018–090-f-S) approved the study.

### 2.2 Memory fMRI-paradigm

The memory fMRI-paradigm comprised scenes, faces and words (see Doll et al., 2021). Scenes were coloured complex pictures mostly taken from the International Affective Picture System (IAPS) set, faces were front-view colour photographs of adult faces, and words were single nouns. Each stimulus condition included 36 randomly selected stimuli with negative and 36 with neutral valence. In three randomised consecutive runs, the stimuli of each condition were presented for 3 s each in alternating blocks of four neutral or four negative stimuli followed by a 12 s baseline condition. Participants were instructed to memorise the stimuli for subsequent recognition. In the baseline condition, participants were requested to maintain fixation at a randomly moving dot and to avoid thinking about the preceding stimuli. Then an out-of-scanner recognition task immediately followed the fMRI-task.

### 2.3 MRI acquisition and pre-processing

MRI data were collected on a 3 T Siemens Verio MRI scanner. For fMRI, 37 coronal slices aligned with the long axis of the hippocampus were acquired. We pre-processed MRI data using fMRIPrep 1.4.1rc4 (Esteban et al., 2019). For further details on anatomical and functional MRI data acquisition and pre-processing see Doll et al. (2021).

### 2.4 Functional connectivity analysis

All analyses were performed using CONN (Whitfield-Gabrieli and Nieto-Castanon, 2012) (RRID:SCR_009550) release 22.a (Nieto-Castanon and Whitfield-Gabrieli, 2022) and SPM (Friston et al., 2011) (RRID:SCR_007037) release 12.7771.

Functional data, which were smoothed with a Gaussian kernel of 8 mm full-width at half-maximum, were denoised using a standard denoising pipeline (Nieto-Castanon, 2020) including the regression of potential confounding effects characterized by white matter and cerebrospinal fluid timeseries (each 6 CompCor noise components), motion linear and quadratic parameters (12 factors) (Friston et al., 1996), task effects (6 factors), and linear trends (2 factors) followed by bandpass frequency filtering of the blood oxygen level dependent (BOLD) timeseries (Hallquist et al., 2013) between 0.008 Hz and 0.1 Hz (Mascali et al., 2021). CompCor noise components (Behzadi et al., 2007, Chai et al., 2012) within white matter and cerebrospinal fluid were estimated by computing the average BOLD signal as well as the largest principal components orthogonal to the BOLD average and motion parameters within each subject's eroded segmentation masks. Afterwards, FC distributions were centered around r = 0.004, and had low variability (SD = 0.002 across participants). Also, associations between quality control measures and FC showed > 95 % overlap with the null hypothesis (quality check parameters: InvalidScans [based on liberal thresholds: global signal change SD = 9 and framewise displacement = 2], MaxGSchange, MaxMotion, MeanGSchange and MeanMotion).

For the first level analysis, seed-based FC maps were estimated with the left and right anterior hippocampus (LAH and RAH) as seed regions. We focused on the anterior hippocampus, because it is especially relevant for episodic encoding and because it has stronger interaction to frontal regions than the posterior hippocampus (Persson et al., 2018). Further, the anterior hippocampus is more often disrupted in mTLE patients than the posterior one (Sone et al., 2022, Bernasconi et al., 2003). We used hippocampus masks defined by the Harvard-Oxford Atlas, which were segmented along the long-axis of the hippocampus. Hippocampus at y ≥ -21 (MNI space), incorporating the uncal apex, was assigned as the anterior hippocampus according to previous literature, see Fig. S1 (Poppenk et al., 2013, Li et al., 2018). FC strength was represented by Fisher-transformed bivariate correlation coefficients from a weighted general linear model (wGLM) (Nieto-Castanon, 2020). Individual scans were weighted by a boxcar function characterizing the three stimulus categories scenes, faces and words and the baseline condition each convolved with an SPM canonical hemodynamic response function and rectified. Following this, we used the CONN Toolbox to calculate both, *abs*-FC during one single condition (e.g. during encoding condition or during baseline) and *rel*-FC changes (FC during encoding compared to baseline), similar to what is done in PPI analysis. The presently used terminology follows the conventions suggested by the CONN toolbox, see (Poletti et al., 2018, Belleau et al., 2020, Sequeira et al., 2021). Elsewhere, *abs*-FC is referred to as “task-state FC” and *rel*-FC is referred to as “task-modulated FC” (Masharipov et al., 2024). More precisely, *abs*-FC is a non-parametric estimation of task-specific connectivity effects. Weighted correlation measures are computed for each condition by weighting the scans associated with that condition only e.g. *abs*-FC scenes = wGLM(“condition scenes”), whilst *rel*-FC represents the difference between two experimental conditions or an experimental condition and its baseline, e.g. *rel*-FC scenes = wGLM(“condition scenes”)-wGLM(“baseline”). In group comparison analysis *abs*-FC can reveal group differences due to alterations in intrinsic or in task-specific connectivity. *rel*-FC instead reveals group differences either specific to the encoding condition only, to the baseline condition only or, in the case of opposing effects during encoding and baseline, a mixture of both. Second level within group and between group comparison analysis were performed using a GLM (Nieto-Castanon, 2020). Results were thresholded using a combination of a cluster-forming threshold of *p* < 0.005 at voxel-level and a familywise corrected cluster-size threshold of *p*-false-discovery-rate (FDR) < 0.05 (Chumbley et al., 2010).

## 3 Results

### 3.1 Controls

**Fig. 1:**
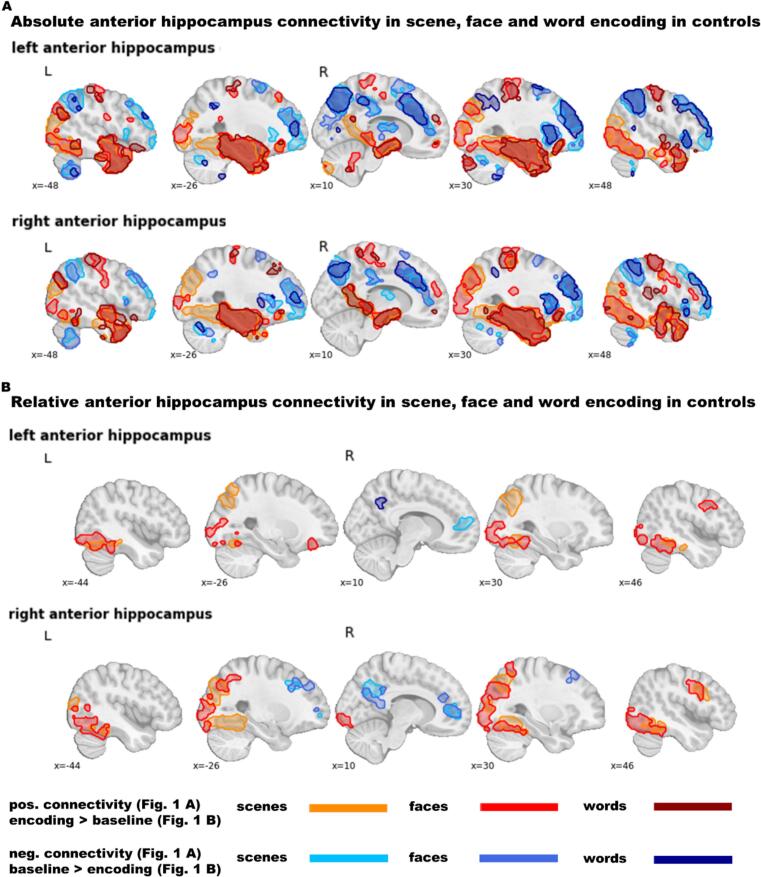
Functional encoding-related connectivity of the left and right anterior hippocampus in controls. **A** Absolute connectivity separately for scene, face, and word encoding. **B** Relative connectivity separately for scene, face, and word encoding compared to baseline. The colour code indicates significant clusters at *p*_(voxel)_ ≤ 0.005 and *p*_(ClusterFDR)_ ≤ 0.05. Data are displayed in MNI-152 space.

**Absolute FC:** As shown in Fig. 1A and Table S1, in controls LAH and RAH *abs*-FC were widespread during encoding of scenes, faces and words. Positive *abs*-FC during tasks spread towards mesio-temporal, lateral temporal, frontal, parietal and occipital regions. Thereby the anterior hippocampus coactivated most strongly with the ipsilateral mTL, followed by the contralateral mTL. Stimulus materials differed in that there was no coactivation to the occipital lobe during word encoding, in contrast to face and scene encoding. Further, coactivation was more widespread during scene encoding in the temporo-occipital fusiform and lingual gyrus and during word and face encoding in the precentral and postcentral gyrus. Finally, only during face encoding did the LAH and RAH coactivate with the ipsilateral inferior frontal gyrus. Negative hippocampus *abs*-FC during tasks was predominantly found in the frontal pole, middle and superior frontal gyrus, paracingulate gyrus, orbitofrontal cortex, insular, frontal operculum, basal ganglia, precuneus, posterior cingulate gyrus, angular gyrus, cuneal cortex, and supramarginal gyrus.

**Relative FC:** The *rel*-FC patterns, contrasting the encoding condition with the baseline, were more circumscribed, see Fig. 1B and Table S2. Seeding from LAH or RAH, *rel*-FC during scene and face encoding was increased in bilateral temporo-occipital regions compared to baseline. These tended to spread more medially during scene and more laterally during face encoding. Additionally, during face encoding, LAH coactivated positively with one cluster encompassing the left frontal pole and one encompassing the right precentral and middle frontal gyrus. Seeding from the RAH, increased *rel*-FC was further present during scene and face encoding in the right precentral and inferior frontal gyrus. During word encoding there was no region with higher *rel*-FC during encoding than baseline. To increase power, we additionally explored the across-group results (across controls, mTLE and FLE). In this large sample the LAH coactivated during word encoding with left frontal regions including the middle and inferior frontal gyrus, the frontal pole, the frontal orbital cortex and the frontal operculum and with small clusters in the left inferior temporal gyrus and lateral occipital cortex. Decreased *rel*-FC during tasks was predominantly found in regions associated with DMN.

### 3.2 Group comparison of absolute FC

**Fig. 2:**
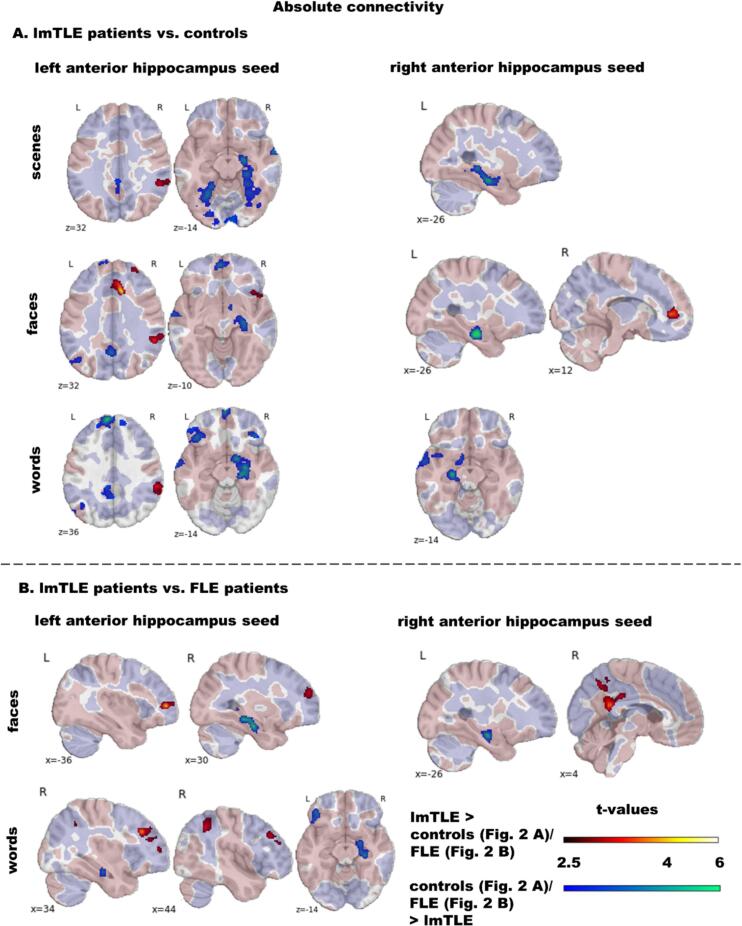
Group comparison of absolute functional encoding-related connectivity of the left and right anterior hippocampus comparing lmTLE patients with **A** controls and **B** FLE patients. We present group comparisons only for stimulus material with significant differences. The colour code indicates significant clusters at *p*_(voxel)_ ≤ 0.005 and *p*_(ClusterFDR)_ ≤ 0.05 during scene, face and word encoding. Additionally, in transparent colours across-group absolute connectivity at t > 0.5 is depicted to demonstrate if the hippocampus in general connects positively (salmon-pink coloured) or negatively (purple-blue coloured) during the encoding condition in clusters with significant group differences. Abbreviations: lmTLE, left mesial temporal lobe epilepsy; FLE, frontal lobe epilepsy. (For interpretation of the references to colour in this figure legend, the reader is referred to the web version of this article.)

**Fig. 3:**
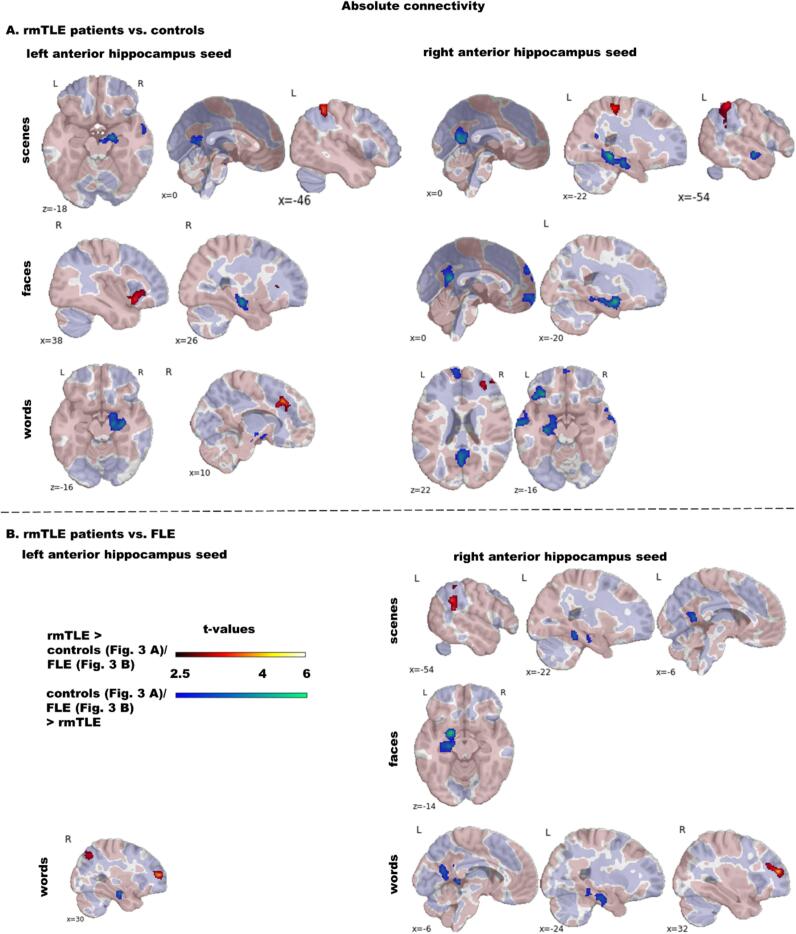
Group comparison of absolute functional encoding-related connectivity of the left and right anterior hippocampus comparing rmTLE patients with **A** controls and **B** FLE patients. We present group comparisons only for stimulus material with significant differences. The colour code indicates significant clusters at *p*_(voxel)_ ≤ 0.005 and *p*_(ClusterFDR)_ ≤ 0.05 during scene, face and word encoding. Additionally, in transparent colours across-group absolute connectivity at t > 0.5 is depicted to demonstrate if the hippocampus in general connects positively (salmon-pink coloured) or negatively (purple-blue coloured) during the encoding condition in clusters with significant group differences. Abbreviations: rmTLE, right mesial temporal lobe epilepsy; FLE, frontal lobe epilepsy. (For interpretation of the references to colour in this figure legend, the reader is referred to the web version of this article.)

Fig. 2, Fig. 3 show that lmTLE and rmTLE patients in comparison to controls and FLE patients had reduced hippocampus *abs*-FC predominantly in regions with overall positive *abs*-FC, and increased *abs*-FC predominantly in regions with overall negative *abs*-FC, see also Tables S3–6. Additionally, Fig. S3-5 depict within-group effects in lmTLE, rmTLE and FLE patients.

**lmTLE patients vs. controls:**Fig. 2 and Table S3 show that lmTLE patients had weaker LAH and to a lesser degree also RAH *abs*-FC than controls. During all stimulus conditions regions of reduced LAH and RAH *abs*-FC encompassed the contralateral mTL. Further, only LAH *abs*-FC was reduced in the precuneus and posterior cingulate gyrus. There were also material-specific changes. During scene encoding LAH *abs*-FC was weaker in widespread bilateral temporo-occipital regions. During face encoding LAH *abs*-FC was reduced in a more circumscribed left temporo-occipital region, the frontal pole and the frontal medial cortex. During word encoding LAH and RAH *abs*-FC was weaker in circumscribed left lateral temporal and occipital regions and only LAH *abs*-FC was reduced to bilateral clusters in the frontal orbital cortex and frontal pole and to a cluster encompassing the frontal pole, superior frontal gyrus and paracingulate gyrus.

Further, lmTLE patients showed regions with stronger hippocampus *abs*-FC than controls during all three conditions. Across materials, these encompassed the supramarginal, and the angular gyrus. For faces, additionally, *abs*-FC was increased to the paracingulate and the anterior cingulate gyrus.

**rmTLE patients vs. controls:** Vice versa to lmTLE, rmTLE patients had weaker RAH and to a lesser degree LAH *abs*-FC than controls. Again, during all conditions LAH and RAH had weaker connectivity to the contralateral mTL. Further, predominantly RAH *abs*-FC to the precuneus and the lingual and posterior cingulate gyrus was reduced. During scene encoding, reduced LAH *abs*-FC to the contralateral mTL extended to the fusiform gyrus. Further, there was weaker LAH *abs*-FC to a right temporo-lateral cluster and weaker RAH *abs*-FC to a left temporo-lateral cluster. During face encoding RAH *abs*-FC was additionally reduced to clusters encompassing the frontal pole, frontal medial cortex and superior frontal gyrus. During word encoding the RAH connected less with bilateral lateral temporal regions, the frontal pole, the superior frontal gyrus and the frontal medial cortex and the left frontal orbital and temporal fusiform cortex.

Regions with increased *abs*-FC in rmTLE patients than controls encompassed again predominantly regions with overall negative task *abs*-FC.

**FLE patients vs. controls**: There were no significant differences in LAH or RAH *abs*-FC between FLE patients and controls.

**lmTLE and rmTLE patients vs. FLE patients**: Direct comparison of lmTLE and rmTLE with FLE patients revealed fewer group differences than the comparison of mTLE patients with controls, see Fig. 2, Fig. 3 and Tables S5 and S6. Reduced *abs*-FC in mTLE patients was predominantly found from the ipsilateral hippocampus to the contralateral mTL. In detail, lmTLE patients had decreased LAH *abs*-FC to the right mTL during face and word encoding. Decreased RAH *abs*-FC to the left mTL was found only during face encoding. Further, during word encoding, LAH *abs*-FC was reduced to a left frontal cluster including the frontal orbital cortex, inferior frontal gyrus and frontal pole. rmTLE patients had decreased RAH *abs*-FC to the left mTL in all three condition and decreased LAH *abs*-FC to the right mTL only during word encoding. Further, RAH *abs*-FC was reduced to the precuneus and posterior cingulate gyrus during scene and word encoding.

Increased *abs*-FC in mTLE patients was again present in regions associated with overall negative *abs*-FC including the frontal pole and middle frontal gyrus, the posterior cingulate gyrus, the precuneus, the angular and supramarginal gyrus and the lateral occipital cortex.

### 3.3 Group comparison of relative FC

**Fig. 4:**
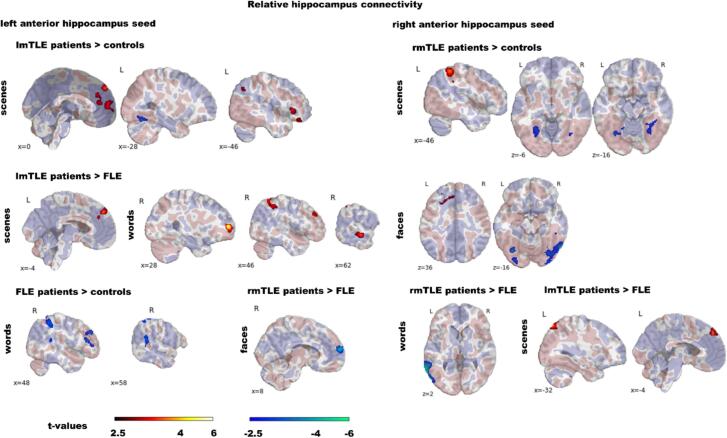
Group comparison of relative functional encoding-related connectivity of the left and right anterior hippocampus comparing lmTLE, rmTLE and FLE patients and controls. We present group comparisons only for stimulus material with significant differences. The colour code indicates significant clusters at *p*_(voxel)_ ≤ 0.005 and *p*_(ClusterFDR)_ ≤ 0.05 during scene, face and word encoding compared to baseline. Additionally, in transparent colours across-group relative connectivity at t > 0.5 is depicted to demonstrate if the hippocampus in general elicits increased (salmon-pink coloured) or decreased (purple-blue coloured) connectivity during the encoding condition compared to baseline in clusters with significant group differences. Abbreviations: FLE, frontal lobe epilepsy; lmTLE, left mesial temporal lobe epilepsy; rmTLE, right mesial temporal lobe epilepsy. (For interpretation of the references to colour in this figure legend, the reader is referred to the web version of this article.)

Fig. 4 and Table S7 show that *rel*-FC in mTLE and FLE patients compared to controls and in mTLE patients compared to FLE patients was predominantly reduced in regions with overall stronger *rel*-FC and increased in regions with overall weaker *rel*-FC during the encoding condition than during baseline. In general, group differences in *rel*-FC were more circumscribed compared to group differences in *abs*-FC.

**lmTLE patients vs. controls:***rel*-FC in lmTLE patients differed from controls only during scene encoding. The LAH was less connected to the left fusiform gyrus and stronger to regions which showed predominantly greater LAH *rel*-FC during baseline than during encoding including the frontal pole, paracingulate, anterior cingulate, superior and inferior frontal and angular gyrus and the frontal orbital cortex.

**rmTLE patients vs. controls:***rel*-FC in rmTLE patients differed from controls during scene and face encoding when seeding from the RAH. During both conditions the RAH connected less to bilateral temporo-occipital regions. Further, patients showed stronger *rel*-FC to the supramarginal and angular gyrus and the superior parietal lobule during scene encoding and to the superior frontal, paracingulate and middle frontal gyrus and frontal pole during face encoding.

**FLE patients vs. controls**: FLE patients had reduced *rel*-FC only during word encoding. The LAH connected less to right-sided clusters encompassing the supramarginal and angular gyrus, the lateral occipital cortex, the middle temporal lobe, the middle and inferior frontal gyrus and the frontal pole.

**lmTLE and rmTLE patients vs. FLE patients**: lmTLE patients had only stronger *rel*-FC than FLE patients. During scene encoding, seeding from either LAH or RAH, lmTLE patients had stronger *rel*-FC to the frontal pole and superior frontal gyrus. Further, RAH *rel*-FC to the left lateral occipital cortex was increased. During word encoding lmTLE patients showed increased LAH *rel*-FC to the right angular and supramarginal gyrus, middle frontal and temporal gyrus and the frontal pole. In contrast, rmTLE patients had only weaker *rel*-FC than FLE patients. In detail, during face encoding, there was reduced LAH *rel*-FC to the frontal pole and paracingulate gyrus and during word encoding reduced RAH *rel*-FC to the left middle temporal gyrus and lateral occipital cortex.

## 4 Discussion

We studied task-based anterior hippocampus FC in healthy controls, mTLE, and FLE patients during an fMRI task of encoding scenes, faces and words, using both *abs*- and *rel*-FC. Overall, task-based FC in controls reflected a mixture of intrinsically correlated and anticorrelated brain networks and task-specific FC that differed between stimulus types. mTLE patients showed strong FC disruptions when seeding from the epileptogenic hippocampus, but also slight disruptions when seeding from the contralateral hippocampus. Some of those disruptions pertained to intrinsic FC, regardless of which stimulus material had to be encoded, others regarded stimulus-specific brain regions. FLE patients had fewer disruptions than mTLE patients. However, they did show weaker *rel*-FC than controls during word encoding. In the following, these results will be discussed in detail.

### 4.1 Hippocampus FC during encoding of scenes, faces and words in controls

For all stimulus types, healthy controls exhibited widespread *abs*-FC from both LAH and RAH. Positive task-based hippocampus *abs*-FC spread towards bilateral frontal, mesio-temporal, temporo-occipital and occipital regions, which largely overlapped with patterns reported in traditional task-based fMRI analysis of memory encoding (Doll et al., 2024, Kim, 2011, Spaniol et al., 2009, Kim, 2024, Golby et al., 2001, Kelley et al., 1998, Cabeza and Nyberg, 2000). In addition, we found *abs*-FC to the superior and middle temporal gyrus, the temporal pole, the ventromedial prefrontal cortex, the precuneus, the posterior cingulate gyrus and the subcallosal cortex. These are regions to which the hippocampus has intrinsic FC regardless of any task (Blessing et al., 2016, Vincent et al., 2006). Many of these regions are also part of the DMN, which aligns with studies finding positive associations between the hippocampus and the DMN (Blessing et al., 2016, Vincent et al., 2006, Ezama et al., 2021) or conceiving of the hippocampus as part of the DMN (Raichle, 2015). The *abs*-FC pattern in controls also overlaps with studies by McCormick et al. (2010) and Foster et al. (2016), who analysed *abs*-FC in healthy people for faces and words, respectively. In our study, patterns are more widespread, probably due to our larger sample size. Stimulus materials differed in that particularly scenes and to a lesser degree also faces elicited *abs*-FC to the occipital lobe, in line with the idea of encoding specificity and stronger perceptual processing of pictorial than verbal material during memory encoding (Kim, 2024, Vaidya et al., 2002). This was not observed for words, probably due to less visual input and their reduced perceptual and enhanced conceptual processing (Kim, 2024). Accordingly, hippocampal *abs*-FC for words was strong to extended bilateral TL areas. Moreover, *abs*-FC to pre- and postcentral regions was most prominent during word encoding. This might reflect embodied language processing (Hauk et al., 2004), encoding-associated tongue or mouth movements (Grabski et al., 2012), or both.

*Rel*-FC from LAH and RAH was more circumscribed than *abs*-FC but likewise present in temporo-occipital regions for scenes and faces. The materials differed especially in face- and scene-selective areas. Faces elicited increased *rel*-FC to the occipital face area (Gauthier et al., 2000), whilst scenes elicited increased *rel*-FC to the parahippocampal (Epstein and Kanwisher, 1998) and occipital place areas (Dilks et al., 2013). In frontal regions we found increased *rel*-FC from LAH and RAH during face encoding. During scene encoding the same was observed solely from RAH. During word encoding there were no regions with stronger *rel*-FC in the controls sample. When we increased power by collapsing results across all groups (see Fig. S2), LAH coactivated especially with left frontal regions as might be theoretically expected (Binding et al., 2022). Accordingly, Schott et al. (2013) reported widespread hippocampus-frontal connectivity in a large group of 64 controls, whereas Fleury et al. (Fleury et al., 2022) found only a very small left frontal cluster in a control group of comparable size to ours, suggesting that *rel*-FC from the hippocampus to inferior frontal gyrus is variable and requires large samples. Moreover, it might be masked by continuous intrinsic connectivity. Rel-FC within or between the hippocampus was not increased in any of the encoding conditions. This is in line with a large study on *rel*-FC during face encoding in older controls (Beason-Held et al., 2021) but contrasts with the study of Fleury et al. (2022), which reports for controls and mTLE patients increased within and between hippocampus *rel*-FC. Maybe the amount of within and between hippocampus FC during baseline periods differs between paradigms. Generally it is well known that there is also high inter- and intra-hippocampus FC during rest (Raichle, 2015, Ma et al., 2022, Seitzman et al., 2019). Further, literature on hippocampal replay suggests that baseline “rest” periods in encoding paradigms are often periods of ongoing covert consolidation with reactivation of hippocampal encoding patterns (Tambini and Davachi, 2013, Foster and Wilson, 2006) underscoring the possibility that *abs*-FC within or between the hippocampi during encoding continues into the baseline, masking this effect in the *rel*-FC analysis. In our study at least, inter- and intra-hippocampus *abs*-FC is high, both during task and baseline, whereas the same is not true for *rel*-FC.

Negative hippocampus *abs*-FC was primarily found in regions largely overlapping with the ventral attention network and the fronto-parietal control network (Yeo et al., 2011). In line with this, previous studies found anticorrelations between these networks and the DMN – to which the hippocampus can be assigned (Zhou et al., 2018, Chen et al., 2017, Martinez-Gutierrez et al., 2022). In addition, negative *rel*-FC showed that the hippocampus connected less to DMN regions during the encoding condition than baseline. This aligns with theory labelling the DMN also the “task-negative network”, because it is expected to be less active during tasks than rest (Fox et al., 2005). Specifically regarding memory encoding, results are also in line with the encoding retrieval flip theory, which assumes that the hippocampus is functionally connected to the DMN during retrieval but decouples during encoding (Huijbers et al., 2012, Huijbers et al., 2011).

So far, we specified typical encoding-related FC in three different commonly used types of visual stimuli, taking into account both task-specific and intrinsic connectivity during encoding (*abs*-FC), and further isolating encoding-specific connectivity by contrasting the encoding condition with the baseline (*rel*-FC). Against this background, we further addressed changes in encoding FC in mTLE and FLE patients who often have lesions in parts of the memory network and exhibit varying degrees of memory disturbances.

### 4.2 FC changes in mTLE patients

mTLE patients had decreased *abs*-FC, which primarily encompassed regions with positive task-based *abs*-FC. Patients differed from controls especially with regard to the epileptogenic hippocampus, which connected less to widespread frontal, temporal, parietal and occipital regions. *Abs*-FC changes from the contralateral hippocampus were more circumscribed and comprised predominantly decreased connectivity to the epileptogenic mTL. In addition, there were some changes from the contralateral hippocampus to extra-mTL regions, which also implicates a slight disruption of the contralateral hippocampus itself. This aligns with resting-state FC studies which report the contralateral mTL to be somewhat disrupted (Pittau et al., 2012, Doucet et al., 2013, Pereira et al., 2010). Across stimuli and independent of epilepsy laterality, the epileptogenic and partly also the contralateral hippocampus displayed less *abs*-FC between the mTLs, the precuneus, and the posterior cingulate gyrus compared to controls. There were no changes in *rel*-FC in these regions. This reveals hippocampus FC to these regions to be generally reduced, both during encoding condition and during baseline. Similar findings exist in resting state studies, also reporting decreased FC between the mTLs in TLE (Pittau et al., 2012, Pereira et al., 2010, de Campos et al., 2016, Li et al., 2015, Li et al., 2017) and between the mTL and the precuneus/posterior cingulate gyrus (de Campos et al., 2016, McCormick et al., 2013, Voets et al., 2014, Haneef et al., 2014, Pittau et al., 2012, Doucet et al., 2013, Pereira et al., 2010). Our results differ from the study by Fleury et al. (2022) investigating *rel*-FC for successful memory formation, where TLE patients showed stronger *rel*-FC (reflected in their PPI analysis) between the mTL than controls. According to our *abs*-FC results, mTLE patients have generally less intrinsic FC between the mTLs than controls during both the encoding condition and baseline, likely contributing to on average worse memory performance. To still achieve successful memory formation, however, some patients might show compensatory mechanisms in terms of increased *rel*-FC between the mTL compared to controls. The fact that Fleury et al. (Fleury et al., 2022) restricted their analysis to subsequently remembered trials may have contributed to this finding in their PPI analysis, which we did not replicate in our *rel*-FC results.

Going beyond resting state studies, task-based FC analysis allowed us to examine material-specific differences during encoding. There were material-specific disruptions in both *abs*-FC and *rel*-FC. During word encoding lmTLE and rmTLE patients alike had reduced *abs*-FC to predominantly left-sided inferior and orbitofrontal regions and the left middle temporal gyrus, concomitant with these regions’ relevance for language processing (Binding et al., 2022). Although we find this effect specifically for words, decrease was only present in *abs*-FC revealing that these changes exist both during encoding and baseline. During scene and face encoding both patient groups had additional disruptions from the epileptogenic hippocampus to predominantly scene- and face-selective temporo-occipital regions, where controls showed strong connectivity. Still, FC decrease to temporo-occipital regions differed between lmTLE and rmTLE patients. Whilst lmTLE showed disruptions primarily in *abs*-FC, implicating decrease both during encoding and during baseline, rmTLE patients had only decreased *rel*-FC. The effects in rmTLE patients arise because these patients display both weaker *abs*-FC to temporo-lateral regions during the encoding condition and, according to additional explorations, stronger *abs*-FC during baseline than controls. This implies that there may be slightly different mechanisms in rmTLE and lmTLE patients. Perhaps rmTLE patients, who are more impaired than lmTLE patients during scene and face encoding (Doll et al., 2021), try to compensate for increased task difficulty by continuing the encoding task during baseline.

Besides FC decrease, there were also regions with increased *abs*-FC in mTLE patients compared to controls. In particular, this applied to regions eliciting negative hippocampus *abs*-FC. Those regions largely overlap with regions of the ventral attention and the fronto-parietal control network. In other words, the functional segregation between the attention and control networks on the one hand and encoding structures on the other seems less strict in patients than in controls, which might hinder task performance. mTLE patients also had stronger hippocampus *rel*-FC than controls to regions of the DMN. Further exploration revealed this to be due to a mixture of less anticorrelated *abs*-FC between the hippocampus and DMN regions during encoding and decreased positive *abs*-FC to DMN regions during baseline. This might hinder performance during the task and could reflect attempts to compensate for increased task difficulty during baseline. Findings align with task-fMRI activation analysis on episodic memory reporting less deactivation in DMN regions in TLE patients compared to controls (Doll et al., 2021, Sidhu et al., 2013).

The direct comparison of mTLE and FLE patients suggests that the FLE patients were overall rather similar to HC. In this contrast, regions with reduced *abs*-FC in mTLE primarily focused on the mTL. Further, lmTLE patients had less LAH *abs*-FC to a left frontal region during word encoding and rmTLE had less RAH *abs*-FC to the precuneus and posterior cingulate gyrus during scene and word encoding. The latter aligns with Bu et al. (Bu et al., 2024) who compared mTLE and FLE patients at rest. Stronger hippocampus *abs*-FC and *rel*-FC was present in similar regions as described above for the comparison to controls. Together, this implicates that many FC changes are specific to a temporal epileptogenic focus, although some common abnormalities in mTLE and FLE patients also exist.

### 4.3 FC changes in FLE patients

Comparison of FLE patients and controls revealed only reduced *rel*-FC during word encoding from the LAH to right-sided regions. These were mainly part of the ventral attention and fronto-parietal control network. To elucidate the origin of these disruptions, we compared FLE and controls also regarding hippocampus *abs*-FC during baseline. *Abs*-FC during baseline was negative in these regions and FLE patients elicited less negative *abs*-FC than controls. Thus, during baseline the segregation between attention and control networks vs. the encoding network seemed less strict in patients. Maybe these changes reflect differences in rehearsal during baseline compared to controls. Finding group differences exclusively for word encoding might be due to contributions of the frontal lobe to language processing. Also, word encoding might benefit more from frontal lobe associated abilities like rehearsal and strategic encoding than face or scene encoding. In line with this, analyses of recognition data of the three conditions showed strongest impairment in FLE patients for words (Doll et al., 2024). The fact that decreased LAH *rel*-FC spread only towards the right hemisphere might be due to sample characteristics, as the sample mainly consisted of right FLE patients. Moreover, our cohort of FLE patients also displayed structural changes in the hippocampus in terms of on average increased left-sided volumes (Doll et al., 2024).

Overall, our results implicate that functional network connectivity during encoding in FLE patients is not as disrupted as in mTLE patients but not equivalent to controls either. This expands existing literature reporting structural and functional activation changes in the mTL in FLE patients (Centeno et al., 2012, Doll et al., 2024, Centeno et al., 2014), which also seem less pronounced than in TLE patients. Such mTL changes, together with impaired orchestration of memory functions by the frontal lobes, might account for memory impairments in FLE patients (Bremm et al., 2019, Exner et al., 2002, Centeno et al., 2012, Cahn-Weiner et al., 2009).

## 5 Strengths and Limitations

To the best of our knowledge, our study is the first to address task-based hippocampus *abs*-FC and *rel*-FC during encoding of different stimulus materials in healthy people as well as mTLE and FLE patients. Comparing mTLE and FLE patients with controls in one study facilitates a specific view on FC changes in epilepsy patients. Investigating both *abs*-FC and *rel*-FC allows for a deeper insight to the origin and mechanism of FC disruptions. Data suggest that this might be especially important in memory tasks, because participants might also differ in their activation during baseline. Although our sample sizes were comparable or larger than in similar studies, some inconsistencies might result from a power problem. Also, the sample size of FLE patients did not allow us to address effects of FLE lateralisation or localisation. Thus, the group of FLE patients was more heterogenous in comparison to mTLE patients, which might have obscured group differences.

## 6 Conclusion

The analysis of anterior hippocampus *abs*-FC and *rel*-FC allowed to capture intrinsic as well as task-specific FC during encoding of scenes, faces and words in healthy people as well as FC changes in focal epilepsy patients. The combination of *abs*-FC and *rel*-FC allowed a more detailed view on the source and mechanism of network changes. Further, it helped us to simultaneously delineate several networks and their changes, which might impact memory performance in focal epilepsy patients. In short, we found that mTLE patients had strong disruptions from the epileptogenic anterior hippocampus. However, also the contralateral hippocampus had slightly disrupted FC. FLE patients had less disruptions than mTLE patients. However, they did show significant alterations in *rel*-FC during word encoding. Together, our findings specify some of the network dynamics underlying the memory impairment in focal epilepsy patients. Given that some of these changes might remit following surgical treatment, future work should determine the association of task-based hippocampus FC changes and memory performance, both pre- and postoperatively. In the long run, such knowledge could further inform surgical interventions.

## CRediT authorship contribution statement

**Anna Doll:** Writing – original draft, Visualization, Investigation, Formal analysis, Data curation. **Daniel A. Schlueter:** Formal analysis. **Martin Wegrzyn:** Software, Methodology, Investigation. **Friedrich G. Woermann:** Methodology, Conceptualization. **Kirsten Labudda:** Supervision, Methodology, Investigation, Funding acquisition, Conceptualization. **Christian G. Bien:** Supervision, Methodology, Funding acquisition, Conceptualization. **Johanna Kissler:** Writing – review & editing, Supervision, Conceptualization.

## Funding

This work was supported by the Deutsche Forschungsgemeinschaft (LA 3567/2–1 and BI 1254/9–1).

## Declaration of competing interest

The authors declare that they have no known competing financial interests or personal relationships that could have appeared to influence the work reported in this paper.

## Data Availability

All unthresholded statistical fMRI maps are available on NeuroVault (https://identifiers.org/neurovault.collection:17925). Further data from this study are available from the corresponding author upon reasonable request.
