# Supplementary material for: Encoding-related hippocampus connectivity for scenes, faces, and words: Healthy people compared to people with temporal and frontal lobe epilepsy
Source: Neuroimage Clin. 2025 Apr 12;46:103784. doi: 10.1016/j.nicl.2025.103784 (PMC12023899; doi:10.1016/j.nicl.2025.103784)
Supplement: Supplementary Data 1 [file mmc1.pdf]

Supplementary Material

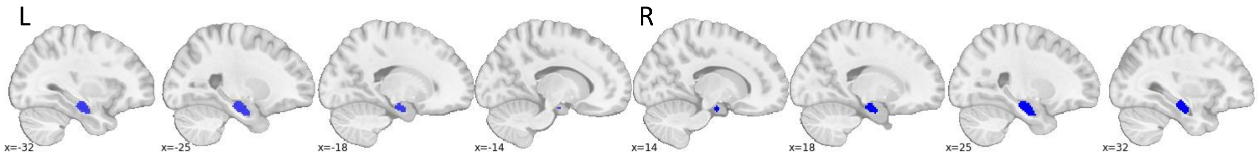

Figure S1. **Left and right anterior hippocampus masks.**

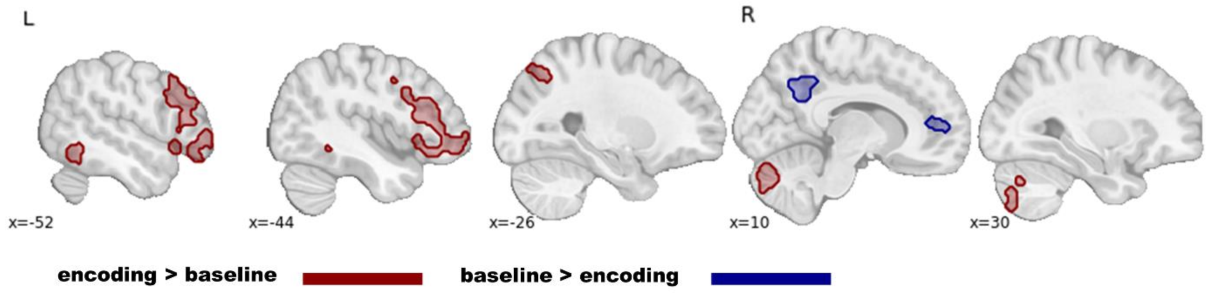

Figure S2. **Across-group relative functional encoding-related connectivity of the left anterior hippocampus during word encoding**. Relative connectivity during word encoding across controls and mTLE and FLE patients. The colour code indicates significant clusters at $p_{(voxel)} \leq .005$ and $p_{(ClusterFDR)} \leq .05$ for all three conditions. Data are displayed in MNI-152 space. . Abbreviations: FLE, frontal lobe epilepsy; mTLE, mesial temporal lobe epilepsy.

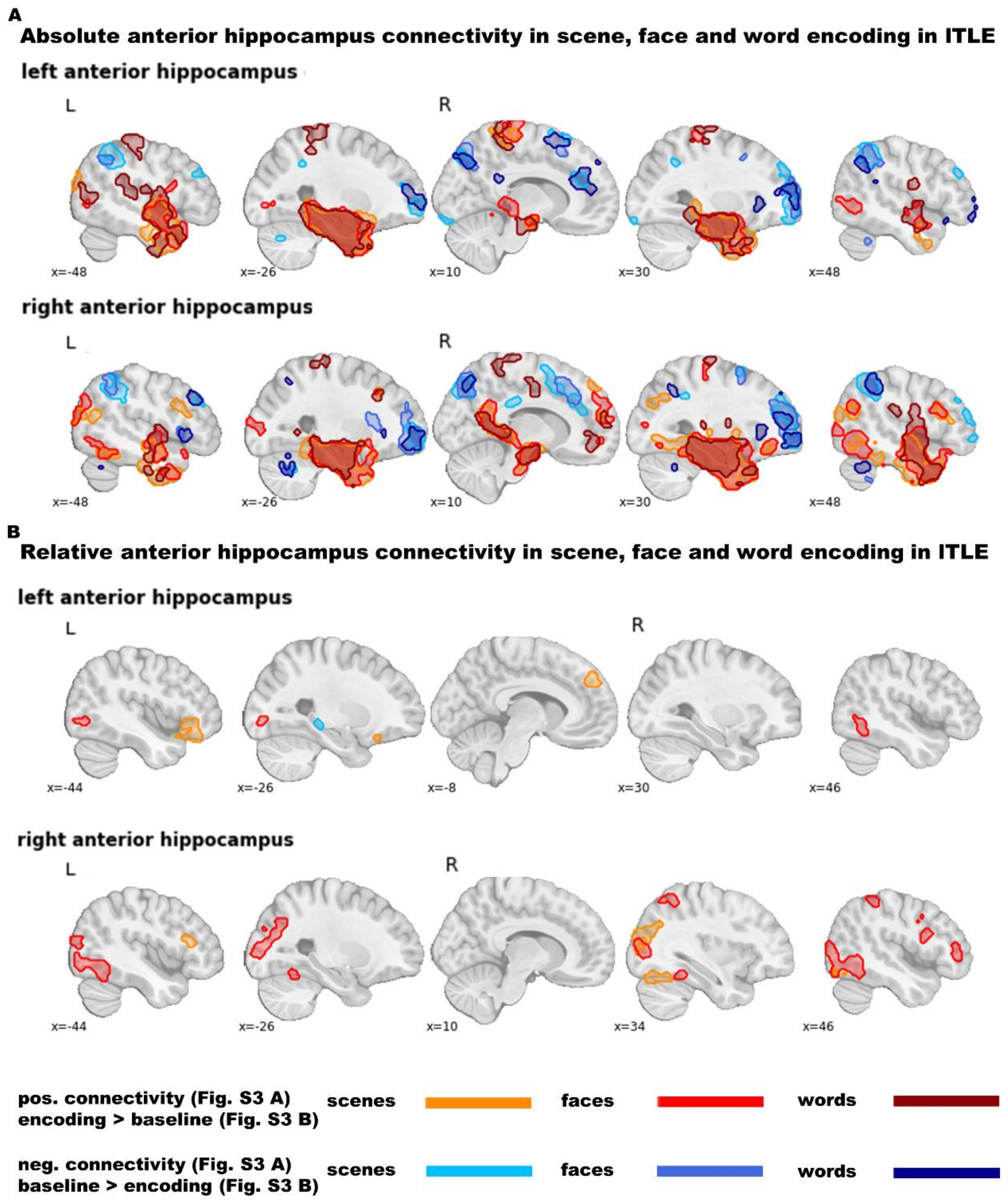

Figure S3. **Functional encoding-related connectivity of the left and right anterior hippocampus in lmTLE**. **A** Absolute connectivity separately for scene, face, and word encoding. **B** Relative connectivity separately for scene and face encoding compared to baseline. We found no significant relative connectivity during word encoding. The colour code indicates significant clusters at $p_{(voxel)} \leq .005$ and $p_{(ClusterFDR)} \leq .05$. Data are displayed in MNI-152 space. Abbreviations: lmTLE, left mesial temporal lobe epilepsy.

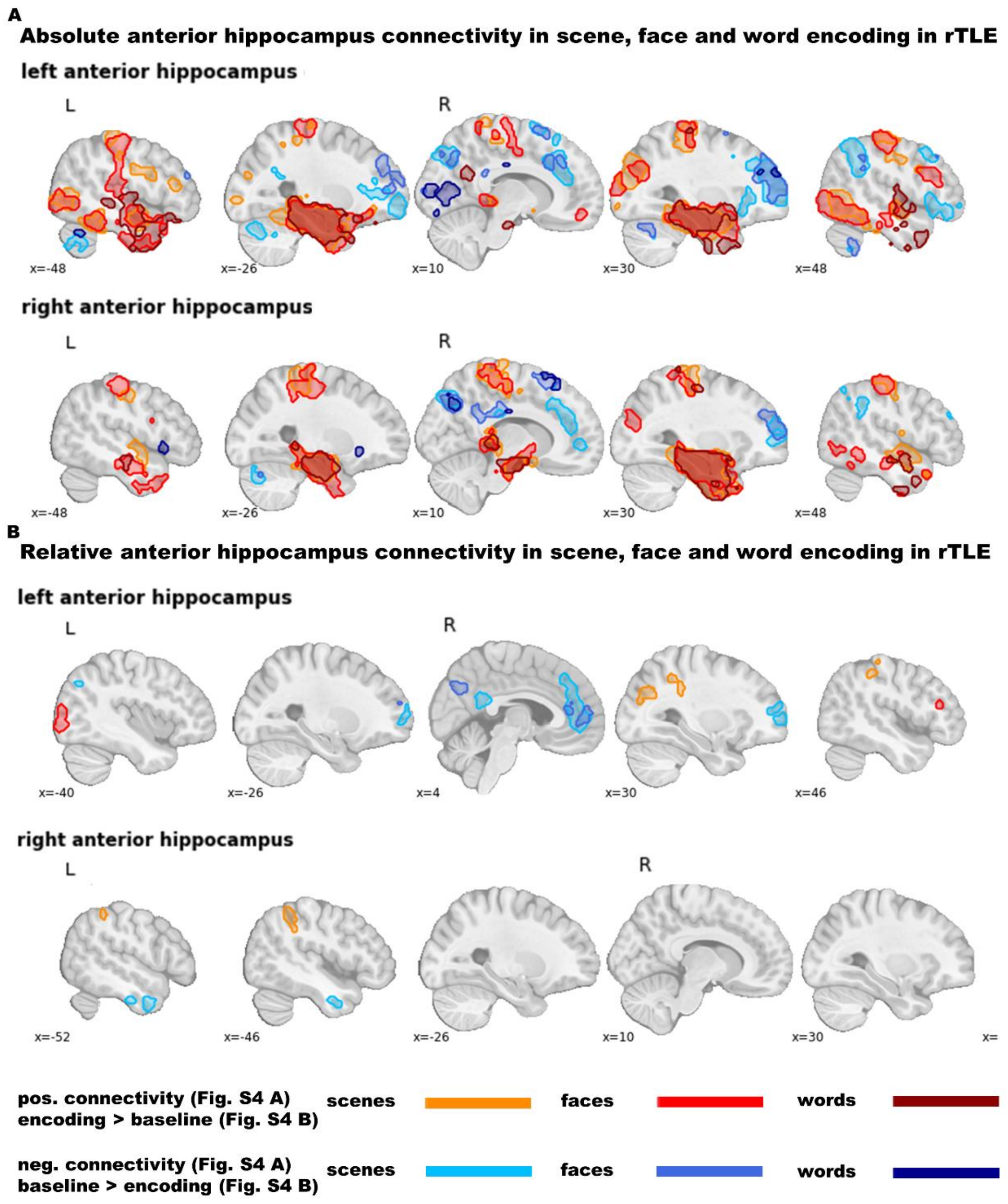

Figure S4. **Functional encoding-related connectivity of the left and right anterior hippocampus in rmTLE**. **A** Absolute connectivity separately for scene, face, and word encoding. **B** Relative connectivity separately for scene and face encoding compared to baseline. We found no significant relative connectivity during word encoding. The colour code indicates significant clusters at $p_{(voxel)} \leq .005$ and $p_{(ClusterFDR)} \leq .05$. Data are displayed in MNI-152 space. Abbreviations: rmTLE, right mesial temporal lobe epilepsy.

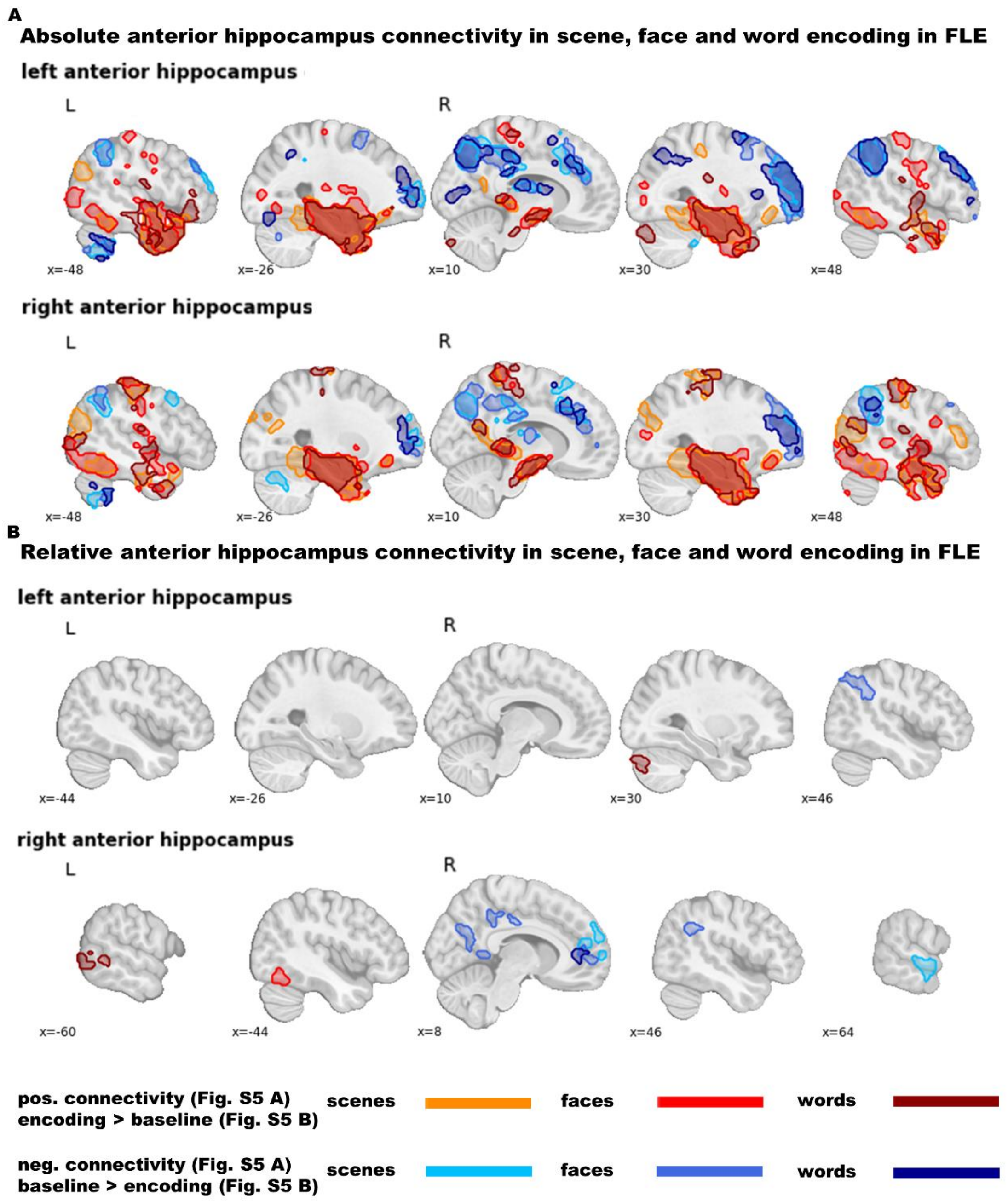

Figure S5. **Functional encoding-related connectivity of the left and right anterior hippocampus in FLE**. **A** Absolute connectivity separately for scene, face, and word encoding. **B** Relative connectivity separately for scene, face, and word encoding compared to baseline. The colour code indicates significant clusters at $p_{(\text{voxel})} \leq .005$ and $p_{(\text{ClusterFDR})} \leq .05$. Data are displayed in MNI-152 space. Abbreviations: FLE, frontal lobe epilepsy.

Table S1

Absolute left and right anterior hippocampus connectivity of healthy controls during scene, face and word encoding.

**Absolute connectivity in healthy controls**					
	Clustersize [mm^3]	Peak	*t*	Peak Region	Cluster Regions [% of cluster in the respective region]
**Scenes: left anterior hippocampus seed**					
**pos.**	203432	-25 -15 -25	38.4	left hippocampus	L: 6.2 temporal pole; 5.8 temporal fusiform cortex; 4.3 hippocampus; 4.3 parahippocampal gyrus; 3.9 middle temporal gyrus; 3.6 inferior temporal gyrus; 3.2 precuneus; 3.1 temporal occipital fusiform; 1.9 amygdala; 1.7 superior temporal gyrus; 1.6 lingual gyrus; 1.2 posterior cingulate gyrus; 1.0 thalamus; 0.9 occipital fusiform gyrus; 0.7 putamen; 0.6 subcallosal cortex; 0.5 lateral occipital cortex; 0.4 accumbens; 0.4 pallidum; 0.4 planum polare; 0.4 insular cortex; 0.2 frontal orbital cortex; 0.2 intracalcarine cortex; 0.1 supracalcarine cortex; 0.1 caudate<br>R: 9.2 lateral occipital cortex; 4.5 temporal fusiform cortex; 4.2 hippocampus; 4.1 parahippocampal gyrus; 4.0 temporal pole; 3.9 temporal occipital fusiform; 3.3 precuneus; 2.8 middle temporal gyrus; 2.3 amygdala; 2.1 inferior temporal gyrus; 1.7 lingual gyrus; 0.8 occipital pole; 0.8 occipital fusiform gyrus; 0.7 posterior cingulate gyrus; 0.7 thalamus; 0.7 superior temporal gyrus; 0.5 subcallosal cortex; 0.4 putamen; 0.3 pallidum; 0.2 accumbens; 0.1 supracalcarine cortex
	16072	-47 -71 24	5.7	left lateral occipital cortex	L: 78.8 lateral occipital cortex; 18.3 occipital pole; 1.8 occipital fusiform gyrus; 0.7 angular gyrus
	5824	-9 56 34	6.8	left frontal pole	L: 66.1 frontal pole; 23.9 superior frontal gyrus<br>R: 7.4 frontal pole; 1.9 superior frontal gyrus
	5696	-3 60 -9	6.9	left frontal pole	L: 25.7 frontal pole; 20.2 frontal medial cortex; 8.0 paracingulate gyrus<br>R: 34.4 frontal pole; 10.4 frontal medial cortex
	3000	-39 36 -17	6.4	left frontal orbital cortex	L: 68.5 frontal orbital cortex; 30.4 frontal pole
	2848	38 36 -17	6.1	right frontal pole	R: 53.7 frontal orbital cortex; 46.1 frontal pole
	1168	24 -103 -9	4.6	right occipital pole	R: 100.0 occipital pole
**neg.**	53096	6 30 40	-8.1	right paracingulate gyrus	L: 10.0 paracingulate gyrus; 2.7 superior frontal gyrus; 2.1 anterior cingulate gyrus<br>R: 41.0 frontal pole; 15.5 middle frontal gyrus; 13.6 paracingulate gyrus; 9.4 superior frontal gyrus; 4.0 anterior cingulate gyrus
	27576	-45 52 16	-8.3	left frontal pole	L: 75.6 frontal pole; 24.2 middle frontal gyrus
	21664	48 -45 46	-11.5	right supramarginal gyrus	R: 46.6 supramarginal gyrus; 34.9 angular gyrus; 12.0 lateral occipital cortex; 4.3 superior parietal lobule; 2.3 parietal operculum
	21048	-11 -73 42	-8.1	left precuneus	L: 29.6 precuneus; 9.7 lateral occipital cortex; 5.2 cuneal cortex<br>R: 37.3 precuneus; 12.3 lateral occipital cortex; 5.5 cuneal cortex
	19056	-57 -47 50	-7.8	left supramarginal gyrus	L: 53.1 supramarginal gyrus; 23.3 angular gyrus; 16.2 lateral occipital cortex; 5.5 superior parietal lobule

	11584	2 -27 28	-9.6	right posterior cingulate gyrus	L: 31.3 posterior cingulate gyrus; 6.8 precuneus; 3.7 precentral gyrus; 2.4 anterior cingulate gyrus<br>R: 37.6 posterior cingulate gyrus; 7.5 precuneus; 3.0 precentral gyrus; 2.6 postcentral gyrus; 1.4 anterior cingulate gyrus
	9440	32 26 6	-7.4	right insular cortex	R: 33.7 insular cortex; 27.8 frontal operculum cortex; 21.4 frontal orbital cortex; 7.5 inferior frontal gyrus pars triangularis; 3.3 inferior frontal gyrus pars opercularis; 3.0 temporal pole; 1.1 frontal pole
	6208	-33 20 10	-7.9	left frontal operculum cortex	L: 40.3 insular cortex; 37.8 frontal operculum cortex; 9.7 frontal orbital cortex; 5.4 inferior frontal gyrus pars opercularis; 3.9 central opercular cortex; 2.8 inferior frontal gyrus pars triangularis
	3504	-17 4 46	-4.2	no label	L: 68.0 superior frontal gyrus; 8.7 middle frontal gyrus
	1512	2 -87 14	-4.2	no label	L: 25.9 intracalcarine cortex; 16.4 cuneal cortex; 7.9 supracalcarine cortex; 5.8 occipital pole<br>R: 18.0 cuneal cortex; 14.3 occipital pole; 9.5 supracalcarine cortex
	1280	14 -15 16	-4.7	right thalamus	R: 92.5 thalamus; 5.6 caudate
**Scenes: right anterior hippocampus seed**					
**pos.**	217336	24 -17 -21	31.0	right hippocampus	L: 4.7 temporal fusiform cortex; 4.2 temporal pole; 3.8 hippocampus; 3.8 parahippocampal gyrus; 3.4 middle temporal gyrus; 2.8 temporal occipital fusiform; 2.8 precuneus; 2.1 lingual gyrus; 2.0 amygdala; 1.8 inferior temporal gyrus; 1.5 occipital fusiform gyrus; 1.4 superior temporal gyrus; 1.0 thalamus; 0.9 posterior cingulate gyrus; 0.8 lateral occipital cortex; 0.5 subcallosal cortex; 0.4 accumbens; 0.4 putamen; 0.4 planum polare; 0.3 insular cortex; 0.3 pallidum; 0.2 intracalcarine cortex; 0.1 supracalcarine cortex; 0.1 caudate; 0.1 frontal orbital cortex; 0.1 occipital pole<br>R: 10.5 lateral occipital cortex; 5.3 temporal pole; 4.9 temporal fusiform cortex; 4.1 hippocampus; 4.0 parahippocampal gyrus; 3.9 temporal occipital fusiform; 3.0 middle temporal gyrus; 2.9 precuneus; 2.7 inferior temporal gyrus; 2.2 lingual gyrus; 2.2 amygdala; 1.5 occipital fusiform gyrus; 1.0 superior temporal gyrus; 0.9 thalamus; 0.8 frontal orbital cortex; 0.8 putamen; 0.7 posterior cingulate gyrus; 0.7 planum polare; 0.5 frontal pole; 0.5 pallidum; 0.5 occipital pole; 0.4 subcallosal cortex; 0.3 insular cortex; 0.3 accumbens; 0.2 intracalcarine cortex; 0.1 supracalcarine cortex
	13448	-43 -79 22	5.2	left lateral occipital cortex	L: 87.6 lateral occipital cortex; 11.9 occipital pole; 0.4 cuneal cortex
	3728	-1 60 -9	5.6	right frontal pole	L: 29.8 frontal pole; 19.3 frontal medial cortex; 3.0 paracingulate gyrus<br>R: 31.3 frontal pole; 16.3 frontal medial cortex
	2232	-37 36 -13	6.5	left frontal orbital cortex	L: 62.4 frontal orbital cortex; 37.6 frontal pole
	1872	54 -15 32	4.6	right postcentral gyrus	R: 84.6 postcentral gyrus; 15.4 precentral gyrus
	1616	-11 54 32	6.0	left frontal pole	L: 70.3 frontal pole; 29.7 superior frontal gyrus
	1608	30 -25 56	5.2	right precentral gyrus	R: 57.2 precentral gyrus; 42.3 postcentral gyrus
	72	-53 -75 4	3.2	left lateral occipital cortex	L: 100.0 lateral occipital cortex
**neg.**	29856	-41 50 14	-7.1	left frontal pole	L: 66.1 frontal pole; 15.5 middle frontal gyrus; 7.5 insular cortex; 5.6 frontal operculum cortex; 3.5 frontal orbital cortex; 0.8 inferior frontal gyrus pars

					opercularis; 0.4 inferior frontal gyrus pars triangularis; 0.2 central opercular cortex
	28440	30 66 -15	-6.9	right frontal pole	R: 76.2 frontal pole; 22.3 middle frontal gyrus; 1.4 paracingulate gyrus
	18040	56 -41 34	-10.1	right supramarginal gyrus	R: 45.9 supramarginal gyrus; 41.6 angular gyrus; 10.8 lateral occipital cortex; 1.3 superior parietal lobule
	17256	12 -69 40	-8.2	right precuneus	L: 28.1 precuneus; 9.6 lateral occipital cortex; 4.9 cuneal cortex<br>R: 43.0 precuneus; 9.9 lateral occipital cortex; 4.4 cuneal cortex
	16216	-63 -45 38	-9.9	left supramarginal gyrus	L: 55.4 supramarginal gyrus; 27.7 angular gyrus; 11.5 lateral occipital cortex; 4.1 superior parietal lobule
	12312	12 38 30	-6.3	no label	L: 32.9 paracingulate gyrus; 11.4 superior frontal gyrus; 5.0 anterior cingulate gyrus<br>R: 38.2 paracingulate gyrus; 7.2 superior frontal gyrus; 4.3 anterior cingulate gyrus
	9184	-41 -55 -51	-6.0	no label	L: 1.2 occipital fusiform gyrus
	7640	-1 -23 28	-10.4	posterior cingulate gyrus	L: 40.8 posterior cingulate gyrus; 4.1 anterior cingulate gyrus<br>R: 47.8 posterior cingulate gyrus; 2.0 anterior cingulate gyrus; 1.4 precentral gyrus
	5752	50 20 -3	-6.5	no label	R: 29.4 frontal orbital cortex; 28.8 frontal operculum cortex; 23.1 insular cortex; 10.0 inferior frontal gyrus pars opercularis; 6.0 inferior frontal gyrus pars triangularis; 1.8 temporal pole
	1344	12 -9 16	-5.3	right thalamus	R: 64.9 thalamus; 16.7 caudate
	1208	-17 -21 18	-4.3	left thalamus	L: 64.9 thalamus; 15.9 caudate
**Faces: left anterior hippocampus seed**					
**pos.**	206136	-27 -17 -19	35.7	left hippocampus	L: 6.7 temporal pole; 5.3 temporal fusiform cortex; 4.7 lateral occipital cortex; 4.4 hippocampus; 4.2 parahippocampal gyrus; 3.9 middle temporal gyrus; 3.5 inferior temporal gyrus; 2.9 occipital pole; 2.4 precuneus; 2.1 amygdala; 2.0 frontal orbital cortex; 2.0 superior temporal gyrus; 1.6 temporal occipital fusiform; 1.1 planum polare; 1.0 occipital fusiform gyrus; 1.0 posterior cingulate gyrus; 0.9 thalamus; 0.8 putamen; 0.6 frontal pole; 0.6 pallidum; 0.5 insular cortex; 0.5 subcallosal cortex; 0.4 planum temporale; 0.4 lingual gyrus; 0.3 accumbens; 0.1 caudate; 0.1 anterior cingulate gyrus<br>R: 5.4 lateral occipital cortex; 4.3 temporal pole; 3.9 hippocampus; 3.7 parahippocampal gyrus; 3.2 temporal fusiform cortex; 3.0 temporal occipital fusiform; 2.6 middle temporal gyrus; 2.4 occipital pole; 2.3 amygdala; 2.3 inferior temporal gyrus; 0.9 precuneus; 0.9 occipital fusiform gyrus; 0.8 thalamus; 0.8 superior temporal gyrus; 0.7 putamen; 0.6 insular cortex; 0.5 posterior cingulate gyrus; 0.5 pallidum; 0.4 frontal orbital cortex; 0.3 subcallosal cortex; 0.3 lingual gyrus; 0.3 accumbens; 0.1 planum polare
	16288	-5 60 -7	9.1	left frontal pole	L: 49.3 frontal pole; 10.0 frontal medial cortex; 7.5 superior frontal gyrus; 2.5 paracingulate gyrus<br>R: 18.9 frontal pole; 10.2 frontal medial cortex; 1.2 superior frontal gyrus
	14912	26 -21 76	6.2	right precentral gyrus	L: 1.8 precentral gyrus; 0.8 postcentral gyrus<br>R: 50.9 postcentral gyrus; 44.9 precentral gyrus
	8064	-9 -67 -21	7.8	no label	R: 1.4 lingual gyrus
	4368	-49 -21 64	5.3	left postcentral gyrus	L: 58.4 postcentral gyrus; 41.6 precentral gyrus

	3784	-55 -75 30	5.2	no label	L: 98.1 lateral occipital cortex
	3168	36 34 -19	7.5	right frontal orbital cortex	R: 68.4 frontal pole; 31.1 frontal orbital cortex
	1992	-25 36 54	5.1	no label	L: 69.9 superior frontal gyrus; 27.7 frontal pole
	1888	-49 -11 24	4.6	no label	L: 85.2 postcentral gyrus; 14.4 precentral gyrus
	1656	30 -77 36	5.8	right lateral occipital cortex	R: 96.1 lateral occipital cortex; 3.9 occipital pole
	1368	-35 -11 14	5.3	left insular cortex	L: 73.7 insular cortex; 25.7 central opercular cortex
	1192	-57 28 8	4.1	left inferior frontal gyrus pars triangularis	L: 69.1 inferior frontal gyrus pars triangularis; 30.9 inferior frontal gyrus pars opercularis
	1072	60 2 26	4.2	right precentral	R: 79.9 precentral gyrus; 20.2 postcentral gyrus
**neg.**	44952	6 42 24	-9.5	right paracingulate gyrus	L: 8.5 paracingulate gyrus; 1.8 superior frontal gyrus; 1.0 anterior cingulate gyrus<br>R: 34.7 frontal pole; 18.5 paracingulate gyrus; 16.3 superior frontal gyrus; 12.7 middle frontal gyrus; 4.5 anterior cingulate gyrus
	43888	-1 -23 30	-13.6	left posterior cingulate gyrus	L: 19.4 precuneus; 7.8 posterior cingulate gyrus; 7.4 lateral occipital cortex; 4.1 cuneal cortex; 2.9 intracalcarine cortex; 2.9 lingual gyrus; 1.1 supracalcarine cortex; 0.8 anterior cingulate gyrus; 0.4 superior parietal lobule; 0.2 occipital fusiform gyrus; 0.2 occipital pole; 0.2 precentral gyrus<br>R: 24.6 precuneus; 11.2 posterior cingulate gyrus; 5.7 lateral occipital cortex; 5.3 cuneal cortex; 1.9 occipital pole; 1.3 supracalcarine cortex; 0.7 lingual gyrus; 0.7 intracalcarine cortex; 0.6 anterior cingulate gyrus; 0.2 precentral gyrus
	17320	60 -39 44	-8.8	right supramarginal gyrus	R: 47.4 supramarginal gyrus; 39.5 angular gyrus; 10.2 lateral occipital cortex; 2.8 superior parietal lobule
	12864	-37 40 24	-5.9	left frontal pole	L: 74.1 frontal pole; 25.9 middle frontal gyrus
	12696	-61 -43 40	-7.0	left supramarginal gyrus	L: 49.7 supramarginal gyrus; 27.4 angular gyrus; 11.5 superior parietal lobule; 9.5 lateral occipital cortex; 1.3 parietal operculum
	9008	40 22 -11	-8.2	right frontal orbital cortex	R: 29.3 frontal orbital cortex; 21.7 insular cortex; 21.1 frontal operculum cortex; 7.9 caudate; 6.0 inferior frontal gyrus pars triangularis; 4.9 temporal pole; 1.8 inferior frontal gyrus pars opercularis
	2792	-17 2 70	-5.1	left superior frontal gyrus	L: 93.4 superior frontal gyrus; 6.3 middle frontal gyrus
	2280	14 -13 12	-5.5	right thalamus	R: 79.3 thalamus; 10.2 pallidum; 7.7 caudate
	1480	22 68 -9	-4.7	right frontal pole	R: 100.0 frontal pole
	1232	-15 -23 18	-6.1	left thalamus	L: 68.8 thalamus; 4.6 caudate
	1224	-35 20 -7	-3.8	left insular cortex	L: 49.0 insular cortex; 26.1 frontal operculum cortex; 24.8 frontal orbital cortex
	304	-13 -35 44	-3.6	left precentral gyrus	L: 42.1 precuneus; 34.2 precentral gyrus; 23.7 posterior cingulate gyrus
**Faces: right anterior hippocampus seed**					
**pos.**	219672	22 -17 -21	35.6	right hippocampus	L: 4.2 temporal pole; 3.8 hippocampus; 3.4 parahippocampal gyrus; 3.4 middle temporal gyrus; 2.8 temporal fusiform cortex; 2.3 lateral occipital cortex; 2.0 amygdala; 1.8 precuneus; 1.8 superior temporal gyrus; 1.1 temporal occipital fusiform; 1.1 occipital pole; 1.0 inferior temporal gyrus; 0.7 postcentral gyrus; 0.7 thalamus; 0.6 subcallosal cortex; 0.6 frontal orbital cortex; 0.6 posterior cingulate gyrus; 0.6 putamen; 0.6 planum polare; 0.5 precentral gyrus; 0.4 occipital fusiform gyrus; 0.4 lingual gyrus; 0.4 accumbens; 0.3 pallidum; 0.1 planum temporale; 0.1 insular cortex; 0.1 caudate

					R: 8.5 lateral occipital cortex; 6.0 postcentral gyrus; 5.4 precentral gyrus; 4.0 hippocampus; 3.9 temporal pole; 3.8 temporal fusiform cortex; 3.7 parahippocampal gyrus; 2.9 middle temporal gyrus; 2.9 inferior temporal gyrus; 2.8 temporal occipital fusiform; 2.3 occipital pole; 2.2 amygdala; 1.6 insular cortex; 1.6 superior temporal gyrus; 1.1 precuneus; 1.1 putamen; 1.0 planum polare; 0.8 thalamus; 0.6 central opercular cortex; 0.6 occipital fusiform gyrus; 0.6 posterior cingulate gyrus; 0.5 lingual gyrus; 0.5 pallidum; 0.3 frontal orbital cortex; 0.2 accumbens; 0.2 subcallosal cortex; 0.2 planum temporale; 0.1 parietal operculum; 0.1 superior parietal lobule; 0.1 superior frontal gyrus
	13736	-35 -21 52	6.2	left precentral gyrus	L: 64.9 postcentral gyrus; 34.7 precentral gyrus
	8504	-9 54 38	9.0	left frontal pole	L: 60.9 frontal pole; 23.0 superior frontal gyrus; 0.2 paracingulate gyrus<br>R: 11.1 frontal pole; 4.7 superior frontal gyrus
	7072	-3 62 -7	6.2	left frontal pole	L: 32.7 frontal pole; 14.6 frontal medial cortex; 1.6 paracingulate gyrus<br>R: 29.9 frontal pole; 20.7 frontal medial cortex;
	2976	-61 -71 22	6.1	no label	L: 97.9 lateral occipital cortex
	2848	36 34 -17	8.0	right frontal orbital cortex	R: 65.5 frontal pole; 34.6 frontal orbital cortex
	2648	-39 36 -17	6.0	left frontal orbital cortex	L: 75.5 frontal orbital cortex; 24.2 frontal pole
	1344	52 34 12	5.2	right inferior frontal gyrus pars triangularis	R: 70.8 inferior frontal gyrus pars triangularis; 27.4 frontal pole; 1.2 middle frontal gyrus
**neg.**	32096	-3 -21 30	-10.5	left posterior cingulate gyrus	L: 26.6 precuneus; 10.8 posterior cingulate gyrus; 8.0 lateral occipital cortex; 3.6 cuneal cortex; 1.0 anterior cingulate gyrus; 0.5 superior parietal lobule<br>R: 26.1 precuneus; 13.4 posterior cingulate gyrus; 5.6 lateral occipital cortex; 2.7 cuneal cortex; 0.8 anterior cingulate gyrus
	18024	-33 48 6	-9.1	left frontal pole	L: 82.3 frontal pole; 17.7 middle frontal gyrus
	15352	4 44 20	-8.3	right paracingulate gyrus	L: 29.0 paracingulate gyrus; 5.8 anterior cingulate gyrus; 5.2 superior frontal gyrus<br>R: 47.3 paracingulate gyrus; 10.5 anterior cingulate gyrus; 2.0 superior frontal gyrus
	13360	62 -43 42	-9.2	right supramarginal gyrus	R: 51.7 supramarginal gyrus; 43.9 angular gyrus; 3.0 lateral occipital cortex; 1.4 superior parietal lobule
	12816	-65 -45 40	-6.8	left supramarginal gyrus	L: 48.3 supramarginal gyrus; 28.4 angular gyrus; 12.5 lateral occipital cortex; 9.2 superior parietal lobule
	12632	28 60 18	-9.7	right frontal pole	R: 89.4 frontal pole; 10.5 middle frontal gyrus
	4816	-37 18 -7	-5.2	left insular cortex	L: 43.7 insular cortex; 28.6 frontal operculum cortex; 15.5 frontal orbital cortex; 4.5 inferior frontal gyrus pars opercularis; 2.3 inferior frontal gyrus pars triangularis
	4640	40 22 -13	-7.1	right frontal orbital cortex	R: 50.0 frontal orbital cortex; 21.7 frontal operculum cortex; 16.6 insular cortex; 6.4 inferior frontal gyrus pars triangularis; 2.9 temporal pole;
	4192	20 12 64	-7.6	right superior frontal gyrus	R: 80.7 superior frontal gyrus; 19.3 middle frontal gyrus
	1880	-23 10 68	-4.9	left superior frontal gyrus	L: 95.7 superior frontal gyrus; 4.3 middle frontal gyrus
	1608	24 68 -9	-5.9	right frontal pole	R: 100 frontal pole
	1112	18 22 8	-5.0	right caudate	R: 79.9 caudate; 6.5 putamen
	88	15 -17 -31	-3.3	no label	L: 72.7 precentral gyrus; 18.2 posterior cingulate gyrus
**Words: left anterior hippocampus seed**					

**pos.**	151096	-25 -15 -23	49.2	left hippocampus	L: 10.0 temporal pole; 6.7 temporal fusiform cortex; 5.8 middle temporal gyrus; 5.5 hippocampus; 5.5 parahippocampal gyrus; 4.5 inferior temporal gyrus; 3.7 frontal orbital cortex; 3.3 precuneus; 2.8 amygdala; 2.2 putamen; 2.2 insular cortex; 2.2 superior temporal gyrus; 1.8 planum polare; 1.7 posterior cingulate gyrus; 1.0 temporal occipital fusiform; 0.9 thalamus; 0.7 pallidum; 0.7 lingual gyrus; 0.6 frontal pole; 0.4 subcallosal cortex; 0.4 accumbens<br>R: 6.7 temporal pole; 4.6 parahippocampal gyrus; 4.5 hippocampus; 3.2 amygdala; 3.1 middle temporal gyrus; 2.5 temporal fusiform cortex; 1.5 superior temporal gyrus; 1.1 putamen; 0.8 frontal orbital cortex; 0.6 precuneus; 0.5 temporal occipital fusiform; 0.5 planum polare; 0.4 pallidum; 0.4 insular cortex; 0.3 lingual gyrus; 0.3 accumbens; 0.3 inferior temporal gyrus; 0.2 subcallosal cortex; 0.2 thalamus
	18656	-9 54 26	8.5	left superior frontal gyrus	L: 58.7 frontal pole; 19.9 superior frontal gyrus; 2.8 frontal medial cortex; 2.1 paracingulate gyrus<br>R: 12.0 frontal pole; 4.0 superior frontal gyrus; 0.4 frontal medial cortex
	9528	32 -27 60	6.2	right postcentral gyrus	R: 59.5 postcentral gyrus; 40.3 precentral gyrus
	5128	36 -81 -37	7.0	no label	R: 8.3 lateral occipital cortex
	4624	-39 -23 62	5.4	left postcentral gyrus	L: 63.8 postcentral gyrus; 36.2 precentral gyrus
	3424	-41 -63 26	4.8	no label	L: 90.0 lateral occipital cortex; 9.6 angular gyrus
	2136	34 34 -17	6.2	right frontal orbital cortex	R: 57.7 frontal orbital cortex; 42.3 frontal pole
	208	50 -15 36	3.9	right postcentral gyrus	R: 100.0 postcentral gyrus
**neg.**	45784	12 -63 54	-8.8	right lateral occipital cortex	L: 15.4 precuneus; 5.5 lateral occipital cortex; 1.0 cuneal cortex; 0.4 posterior cingulate gyrus<br>R: 20.8 precuneus; 20.8 supramarginal gyrus; 14.9 lateral occipital cortex; 12.6 angular gyrus; 4.6 superior parietal lobule; 1.9 posterior cingulate gyrus; 1.6 cuneal cortex; 0.2 postcentral gyrus
	18480	38 48 14	-7.7	right frontal pole	R: 86.5 frontal pole; 12.7 middle frontal gyrus; 0.7 precentral gyrus
	14944	6 26 42	-9.8	right paracingulate gyrus	L: 31.5 paracingulate gyrus; 4.1 superior frontal gyrus; 1.7 anterior cingulate gyrus<br>R: 43.2 paracingulate gyrus; 9.6 anterior cingulate gyrus; 9.3 superior frontal gyrus
	7176	-39 -55 44	-6.6	no label	L: 52.6 supramarginal gyrus; 25.9 angular gyrus; 14.1 superior parietal lobule; 6.9 lateral occipital cortex
	7016	32 24 6	-5.5	right insular cortex	R: 28.9 frontal operculum cortex; 23.7 frontal orbital cortex; 21.8 insular cortex; 16.2 inferior frontal gyrus pars opercularis; 5.0 temporal pole; 2.3 precentral gyrus; 2.2 inferior frontal gyrus pars triangularis
	6240	-45 42 26	-5.4	left frontal pole	L: 92.7 frontal pole; 7.3 middle frontal gyrus
	3192	62 -31 -15	-6.2	right middle temporal gyrus	R: 86.7 middle temporal gyrus; 11.0 inferior temporal gyrus
	2856	-1 -81 -1	-4.7	left lingual gyrus	L: 41.7 intracalcarine cortex; 21.6 lingual gyrus; 8.4 occipital pole; 1.7 supracalcarine cortex<br>R: 13.7 intracalcarine cortex; 12.6 lingual gyrus
	2800	-1 -25 28	-5.9	left posterior cingulate gyrus	L: 59.4 posterior cingulate gyrus; 1.7 anterior cingulate gyrus<br>R: 38.9 posterior cingulate gyrus
	1312	20 16 64	-4.8	right superior frontal gyrus	R: 76.8 superior frontal gyrus; 23.2 middle frontal gyrus

	1128	44 8 46	-4.6	right middle frontal gyrus	R: 75.2 middle frontal gyrus; 24.1 precentral gyrus
**Words: right anterior hippocampus seed**					
**pos.**	166080	22 -13 -21	37.1	right hippocampus	L: 6.8 temporal pole; 5.3 middle temporal gyrus; 4.9 temporal fusiform cortex; 4.8 hippocampus; 4.7 parahippocampal gyrus; 3.3 precuneus; 3.2 inferior temporal gyrus; 2.6 amygdala; 2.4 superior temporal gyrus; 1.3 planum polare; 1.2 posterior cingulate gyrus; 0.9 insular cortex; 0.9 temporal occipital fusiform; 0.7 lingual gyrus; 0.6 frontal orbital cortex; 0.6 thalamus; 0.6 putamen; 0.5 subcallosal cortex; 0.4 accumbens; 0.3 pallidum; 0.1 planum temporale; 0.1 supracalcarine cortex; 0.1 intracalcarine cortex<br>R: 8.6 temporal pole; 5.1 parahippocampal gyrus; 4.9 hippocampus; 4.9 temporal fusiform cortex; 4.8 middle temporal gyrus; 2.9 amygdala; 2.5 superior temporal gyrus; 2.3 precuneus; 2.3 inferior temporal gyrus; 1.9 planum polare; 1.8 temporal occipital fusiform; 1.2 lingual gyrus; 0.8 thalamus; 0.8 insular cortex; 0.8 posterior cingulate gyrus; 0.7 putamen; 0.7 frontal orbital cortex; 0.5 pallidum; 0.4 subcallosal cortex; 0.3 accumbens; 0.1 planum temporale; 0.1 central opercular cortex; 0.1 cuneal cortex
	20248	14 -41 68	5.6	right postcentral gyrus	L: 11.6 precentral gyrus; 7.7 postcentral gyrus<br>R: 51.6 postcentral gyrus; 26.1 precentral gyrus; 2.7 superior parietal lobule
	8328	-19 38 48	5.8	left superior frontal gyrus	L: 67.2 frontal pole; 26.8 superior frontal gyrus; 5.8 middle frontal gyrus
	5408	-51 -31 58	5.2	left postcentral gyrus	L: 89.9 postcentral gyrus; 7.7 precentral gyrus; 1.9 superior parietal lobule
	5008	42 -27 10	5.2	no label	R: 23.8 parietal operculum; 22.4 planum temporale; 16.0 superior temporal gyrus; 9.6 insular cortex; 8.0 central opercular cortex; 3.5 supramarginal gyrus
	4992	-3 66 -11	6.8	left frontal pole	L: 42.2 frontal pole; 15.1 frontal medial cortex; 6.9 paracingulate gyrus<br>R: 31.6 frontal pole; 3.9 frontal medial cortex
	3448	-33 32 -17	6.9	left frontal orbital cortex	L: 71.7 frontal orbital cortex; 24.8 frontal pole; 3.5 temporal pole
	3424	-53 -73 34	4.7	left lateral occipital cortex	L: 93.7 lateral occipital cortex; 5.8 angular gyrus
	1592	40 32 -19	6.0	right frontal orbital cortex	R: 60.8 frontal orbital cortex; 39.2 frontal pole
	1208	-37 -33 12	5.0	left planum temporale	L: 47.0 planum temporale; 11.9 insular cortex; 9.9 parietal operculum;
	1144	38 -79 8	4.4	right lateral occipital cortex	R: 100.0 lateral occipital cortex
	592	-67 -31 12	3.9	left superior temporal gyrus	L: 86.5 superior temporal gyrus; 12.2 planum temporale
**neg.**	12400	38 42 36	-6.8	right frontal pole	R: 87.7 frontal pole; 12.3 middle frontal gyrus
	12136	6 30 38	-7.4	right paracingulate gyrus	L: 25.4 paracingulate gyrus; 6.5 superior frontal gyrus<br>R: 48.0 paracingulate gyrus; 11.5 superior frontal gyrus; 4.8 anterior cingulate gyrus
	9384	58 -33 44	-6.0	right supramarginal gyrus	R: 66.2 supramarginal gyrus; 22.0 angular gyrus; 11.6 superior parietal lobule
	9120	14 -75 44	-6.1	right lateral occipital cortex	L: 32.3 precuneus; 6.5 lateral occipital cortex<br>R: 37.5 precuneus; 21.6 lateral occipital cortex; 1.8 cuneal cortex
	5424	36 22 12	-6.4	right frontal operculum	R: 34.7 frontal operculum cortex; 31.0 frontal orbital cortex; 25.5 insular cortex; 5.3 inferior frontal gyrus

				pars triangularis; 1.8 inferior frontal gyrus pars opercularis
4360	-57 -45 38	-7.1	left supramarginal gyrus	L: 91.9 supramarginal gyrus; 6.6 angular gyrus
3032	-27 56 10	-4.7	left frontal pole	L: 100.0 frontal pole
2712	-39 -55 -33	-5.7	no label	L: 4.7 occipital fusiform gyrus;
1744	-3 -27 28	-5.5	left posterior cingulate gyrus	L: 63.8 posterior cingulate gyrus<br>R: 36.2 posterior cingulate gyrus
1568	-17 12 4	-5.0	no label	L: 67.4 putamen; 28.1 caudate
1472	-41 36 34	-4.3	left middle frontal gyrus	L: 67.9 middle frontal gyrus; 32.1 frontal pole
1352	-39 -55 42	-5.2	no label	L: 62.1 angular gyrus; 21.3 superior parietal lobule; 12.4 lateral occipital cortex
1320	-41 16 10	-4.2	left frontal operculum cortex	L: 77.6 frontal operculum cortex; 17.6 insular cortex

Note. Abbreviations: L, left hemisphere; R, right hemisphere.

Table S2

Relative left and right anterior hippocampus connectivity of healthy controls during scene, face and word encoding.

**Relative connectivity in healthy controls**					
	Clustersize [mm^3]	Peak	*t*	Peak Region	Cluster Regions [% of cluster in the respective region]
**Scenes: left anterior hippocampus seed**					
**pos.**	7216	-37 -63 -21	6.8	left temporal occipital fusiform cortex	L: 43.0 temporal occipital fusiform; 35.0 occipital fusiform gyrus; 8.7 lateral occipital cortex; 8.4 temporal fusiform cortex; 4.5 inferior temporal gyrus
	5592	32 -65 -19	5.6	right occipital fusiform gyrus	R: 37.5 temporal occipital fusiform; 27.3 occipital fusiform gyrus; 17.3 inferior temporal gyrus; 9.4 lateral occipital cortex; 8.0 temporal fusiform cortex
	4616	30 -67 42	5.4	right lateral occipital cortex	R: 98.6 lateral occipital cortex; 1.2 superior parietal lobule
	3928	-29 -75 60	6.1	no label	L: 95.5 lateral occipital cortex; 4.3 superior parietal lobule
**neg.**	6200	12 56 10	-5.4	no label	L: 29.6 paracingulate gyrus; 12.5 frontal pole; 3.0 anterior cingulate gyrus<br>R: 30.5 paracingulate gyrus; 16.9 frontal pole; 6.3 anterior cingulate gyrus
**Scenes: right anterior hippocampus seed**					
**pos.**	24744	-33 -63 -11	7.4	no label	L: 36.8 lateral occipital cortex; 20.3 temporal occipital fusiform; 15.7 occipital fusiform gyrus; 9.0 temporal fusiform cortex; 8.9 lingual gyrus; 7.9 occipital pole; 0.9 inferior temporal gyrus
	10664	34 -57 -19	8.1	right temporal occipital fusiform cortex	R: 50.8 temporal occipital fusiform; 29.0 occipital fusiform gyrus; 7.7 lingual gyrus; 6.2 inferior temporal gyrus; 4.1 lateral occipital cortex; 2.1 temporal fusiform cortex
	5792	30 -69 40	5.2	right lateral occipital cortex	R: 98.1 lateral occipital cortex; 1.8 superior parietal lobule
	3456	46 8 34	5.7	right precentral gyrus	R: 44.2 inferior frontal gyrus pars opercularis; 35.4 precentral gyrus; 19.4 middle frontal gyrus
	2024	34 -89 12	4.0	right occipital pole	R: 50.6 lateral occipital cortex; 49.4 occipital pole
	96	40 -27 -25	3.4	right temporal fusiform cortex	R: 100.0 temporal fusiform cortex
**neg.**	11784	6 -63 36	-5.3	right precuneus	L: 34.4 precuneus; 14.9 posterior cingulate gyrus; 2.7 cuneal cortex<br>R: 35.8 precuneus; 10.7 posterior cingulate gyrus; 1.4 cuneal cortex
	6280	4 44 6	-6.1	right anterior cingulate gyrus	L: 22.2 paracingulate gyrus; 14.1 frontal pole; 4.5 anterior cingulate gyrus<br>R: 32.2 paracingulate gyrus; 14.3 frontal pole; 12.4 anterior cingulate gyrus
	2744	-27 18 40	-5.2	no label	L: 51.9 superior frontal gyrus; 47.8 middle frontal gyrus
	1536	-35 52 10	-4.9	left frontal pole	L: 100.0 frontal pole
**Faces: left anterior hippocampus seed**					
**pos.**	20464	62 -65 -3	5.3	no label	R: 37.1 lateral occipital cortex; 17.4 temporal occipital fusiform; 17.1 inferior temporal gyrus; 16.9 occipital pole; 8.1 occipital fusiform gyrus; 2.4 middle temporal gyrus; 0.8 temporal fusiform cortex
	19256	-47 -65 -9	5.3	left lateral occipital cortex	L: 38.8 lateral occipital cortex; 28.1 occipital pole; 14.8 occipital fusiform gyrus; 12.1 inferior temporal

					gyrus; 5.1 temporal occipital fusiform; 0.8 temporal fusiform cortex
	3040	40 6 38	4.6	right middle frontal gyrus	R: 55.0 precentral gyrus; 44.7 middle frontal gyrus
	3032	-29 38 -19	6.5	left frontal pole	L: 57.8 frontal pole; 24.0 frontal orbital cortex; 17.2 frontal medial cortex
**Faces: right anterior hippocampus seed**					
**pos.**	37960	42 -77 -21	5.7	no label	R: 50.3 lateral occipital cortex; 20.7 occipital pole; 8.5 occipital fusiform gyrus; 8.4 inferior temporal gyrus; 7.6 temporal occipital fusiform; 3.1 middle temporal gyrus; 0.7 superior parietal lobule; 0.5 lingual gyrus
	21624	-39 -71 -19	6.2	left occipital fusiform gyrus	L: 43.2 lateral occipital cortex; 29.3 occipital pole; 12.0 occipital fusiform gyrus; 7.3 inferior temporal gyrus; 5.2 temporal occipital fusiform; 1.0 middle temporal gyrus; 0.9 temporal fusiform cortex
	6768	50 6 34	5.6	right precentral gyrus	R: 74.1 precentral gyrus; 13.8 middle frontal gyrus; 9.8 inferior frontal gyrus pars opercularis; 2.0 postcentral gyrus
	2824	-29 -61 48	5.1	left lateral occipital cortex	L: 84.1 lateral occipital cortex; 15.9 superior parietal lobule
	272	-23 -99 26	3.4	no label	L: 52.9 occipital pole; 44.1 lateral occipital cortex
	88	34 -77 16	3.4	right lateral occipital cortex	R: 100.0 lateral occipital cortex
**neg.**	17832	16 32 24	-6.9	no label	L: 33.1 paracingulate gyrus; 11.0 anterior cingulate gyrus; 8.0 superior frontal gyrus; 4.1 middle frontal gyrus; 3.6 frontal pole<br>R: 24.6 paracingulate gyrus; 10.1 anterior cingulate gyrus; 4.0 frontal pole
	4960	-7 -69 30	-4.8	left precuneus	L: 39.8 precuneus<br>R: 54.4 precuneus; 5.3 posterior cingulate gyrus
	2104	-3 -47 40	-4.3	left precuneus	L: 33.1 posterior cingulate gyrus; 30.0 precuneus<br>R: 31.6 posterior cingulate gyrus; 5.3 precuneus
	1472	28 28 54	-4.6	right superior frontal gyrus	R: 54.9 superior frontal gyrus; 42.9 middle frontal gyrus; 2.2 frontal pole
	728	-9 -47 10	-4.3	left posterior cingulate gyrus	L: 68.1 posterior cingulate gyrus; 16.5 precuneus<br>R: 12.1 precuneus; 3.3 posterior cingulate gyrus
	520	-27 46 42	-3.7	left frontal pole	L: 96.9 frontal pole; 3.1 middle frontal gyrus
**Words: left anterior hippocampus seed**					
**neg.**	5088	2 -61 44	-5.0	right precuneus	L: 46.9 precuneus; 9.9 posterior cingulate gyrus<br>R: 42.9 precuneus

Note. There is no significant relative connectivity for the right anterior hippocampus during word encoding. Abbreviations: L, left hemisphere; R, right hemisphere.

Table S3

Group comparisons of absolute left and right anterior hippocampus connectivity of controls and lmTLE patients during scene, face and word encoding.

**Group comparison: absolute connectivity: lmTLE patients vs. controls**					
	Clustersize [mm^3]	Peak	*t*	Peak Region	Cluster Regions [% of cluster in the respective region]
**Scenes: left anterior hippocampus seed**					
**lmTLE < HC**	15544	36 -29 -25	-5.5	right temporal fusiform cortex	R: 31.8 temporal occipital fusiform cortex; 17.0 temporal fusiform cortex; 14.3 hippocampus; 11.7 occipital fusiform gyrus; 7.3 lingual gyrus; 10.0 parahippocampal gyrus; 3.7 lateral occipital cortex; 2.8 amygdala; 1.5 inferior temporal gyrus
	13528	-13 -51 8	-5.1	left precuneus	L: 22.7 precuneus; 21.8 temporal occipital fusiform cortex; 16.0 posterior cingulate gyrus; 15.4 occipital fusiform gyrus; 7.6 lingual gyrus; 5.8 hippocampus; 1.6 thalamus; 1.6 lateral occipital cortex<br>R: 3.4 precuneus; 2.3 posterior cingulate gyrus
	2360	8 -97 -15	-4.0	no label	L: 35.9 occipital pole; 5.4 lingual gyrus<br>R: 41.4 occipital pole; 16.6 lingual gyrus
	2184	62 2 -29	-4.9	right middle temporal gyrus	R: 89.7 middle temporal gyrus; 5.1 superior temporal gyrus; 4.4 temporal pole
	224	10 -5 -9	-4.0	no label	R: 57.1 pallidum; 10.7 amygdala
**lmTLE > HC**	4576	58 -39 54	5.3	right supramarginal gyrus	R: 73.4 supramarginal gyrus; 25.2 angular gyrus
**Scenes: right anterior hippocampus seed**					
**lmTLE < HC**	4856	-27 -21 -19	-5.3	left hippocampus	L: 70.7 hippocampus; 11.4 lingual gyrus; 5.4 temporal fusiform cortex; 6.3 parahippocampal gyrus; 4.1 amygdala; 2.1 thalamus
**Faces: left anterior hippocampus seed**					
**lmTLE < HC**	6624	20 -13 -23	-6.1	right hippocampus	R: 58.3 hippocampus; 20.3 amygdala; 13.5 parahippocampal gyrus; 2.0 pallidum
	5224	-5 60 -7	-5.2	left frontal pole	L: 77.0 frontal pole; 8.7 frontal medial cortex; 2.3 superior frontal gyrus<br>R: 10.9 frontal pole
	3880	-5 -55 18	-5.0	left precunus	L: 51.6 precuneus; 20.0 cingulate gyrus;<br>R: 25.7 precuneus; 2.7 cingulate gyrus
	2416	-67 -5 -21	-5.9	no label	L: 99.3 middle temporal gyrus
	1832	-59 -67 24	-4.1	left lateral occipital cortex	L: 97.8 lateral occipital cortex
	168	-11 -47 40	-3.2	left precuneus	L: 100 precuneus
**lmTLE > HC**	4784	12 24 32	5.3	right paracingulate gyrus	R: 60.0 paracingulate gyrus; 17.4 anterior cingulate gyrus;<br>L: 22.1 paracingulate gyrus; 1 anterior cingulate gyrus
	3432	64 -39 38	4.8	right supramarginal gyrus	R: 93.7 supramarginal gyrus; 6.3 angular gyrus
	2056	42 20 6	3.6	right frontal operculum	R: 30.0 frontal operculum; 24.1 temporal pole; 13.2 insular; 12.8 frontal orbital cortex; 9.3 precentral gyrus; 6.2 central opercular cortex; 4.3 inferior frontal gyrus pars opercularis
	1888	28 54 28	4.2	right frontal pole	R: 100 frontal pole

**Faces: right anterior hippocampus seed**					
**lmTLE < HC**	3760	-25 -17 -19	-6.5	left hippocampus	L: 71.9 hippocampus; 18.7 amygdala; 9.2 parahippocampal gyrus
**lmTLE > HC**	2256	12 46 4	4.4	right paracingulate gyrus	L: 11.7 paracingulate gyrus; 4.3 anterior cingulate gyrus<br>R: 65.3 paracingulate gyrus; 18.4 anterior cingulate gyrus
	64	-9 46 10	3.1	left paracingulate gyrus	L: 100 paracingulate gyrus
**Words: left anterior hippocampus seed**					
**lmTLE < HC**	20736	-5 56 26	-7.0	left superior frontal gyrus	L: 52.2 frontal pole; 18.9 superior frontal gyrus; 5.0 paracingulate gyrus; 1.7 frontal medial cortex<br>R: 16.4 frontal pole; 4.4 superior frontal gyrus; 1.3 paracingulate gyrus
	7728	28 -19 -19	-6.5	right hippocampus	R: 42.4 hippocampus; 30.9 amygdala; 10.7 parahippocampal gyrus; 4.1 pallidum; 2.4 putamen; 1.8 accumbens
	3616	-43 30 -17	-4.4	left frontal orbital cortex	L: 71.0 frontal orbital cortex; 26.8 frontal pole; 2.2 temporal pole
	1792	-41 -63 28	-3.7	no label	L: 85.3 lateral occipital cortex; 13.8 angular gyrus
	1688	-9 -41 34	-3.7	left posterior cingulate gyrus	L: 69.7 posterior cingulate gyrus; 28.4 precuneus<br>R: 1.4 precuneus
	1632	40 30 -19	-5.6	right frontal orbital cortex	R: 65.2 frontal orbital cortex; 34.3 frontal pole
	1600	-63 -5 -35	-3.8	no label	L: 95.0 middle temporal gyrus; 4.0 inferior temporal gyrus
**lmTLE > HC**	5032	60 -47 50	4.3	no label	R. 81.6 supramarginal gyrus; 18.0 angular gyrus
	2968	-15 -41 52	4.4	left postcentral gyrus	L: 33.7 postcentral gyrus; 27.2 precuneus; 2.4 precentral gyrus; 1.4 superior parietal lobule<br>R: 32.9 precuneus; 1.9 postcentral gyrus
	112	18 -37 48	3.9	right precuneus	R: 64.3 postcentral gyrus; 21.4 precuneus
**Words: right anterior hippocampus seed**					
**lmTLE < HC**	4400	-25 -17 -19	-6.0	left hippocampus	L: 61.6 hippocampus; 16.7 amygdala; 9.1 parahippocampal gyrus; 4.9 accumbens
	2312	-59 -1 -15	-3.9	left middle temporal gyrus	L: 47.1 middle temporal gyrus; 33.2 temporal pole; 12.1 superior temporal gyrus; 6.2 insular cortex

Note. Abbreviations: L, left hemisphere; lmTLE, left mesial temporal lobe epilepsy; HC, healthy controls; R, right hemisphere.

Table S4

Group comparisons of absolute left and right anterior hippocampus connectivity of controls and rmTLE patients during scene, face and word encoding.

**Group comparison: absolute connectivity: rmTLE patients vs. controls**					
	Clustersize [mm³]	Peak	*t*	Peak Region	Cluster Regions [% of cluster in the respective region]
**Scenes: left anterior hippocampus seed**					
**rmTLE < HC**	3936	-3 -55 8	-4.3	left precuneus	L: 67.3 precuneus; 13.0 posterior cingulate gyrus; 5.1 intracalcarine cortex; 3.1 lingual gyrus<br>R: 7.9 precuneus; 2.9 intracalcarine cortex
	2272	18 -11 -19	-5.0	right hippocampus	R: 36.3 hippocampus; 19.4 amygdala; 6.7 pallidum
	1968	60 2 -27	-4.8	right middle temporal gyrus	R: 90.7 middle temporal gyrus; 6.5 temporal pole; 2.9 inferior temporal gyrus
**rmTLE > HC**	2048	-49 -47 58	4.4	left supramarginal gyrus	L: 53.5 superior parietal lobule; 46.5 supramarginal gyrus
**Scenes: right anterior hippocampus seed**					
**rmTLE < HC**	22376	-7 -57 10	-7.2	left precuneus	L: 21.6 precuneus; 10.8 lingual gyrus; 10.2 hippocampus; 7.0 temporal fusiform cortex; 8.2 parahippocampal gyrus; 6.8 temporal occipital fusiform cortex; 4.7 posterior cingulate gyrus; 3.4 inferior temporal gyrus; 3.0 occipital fusiform gyrus; 1.3 thalamus; 1.3 intracalcarine cortex; 0.5 supracalcarine cortex<br>R: 16.5 precuneus; 1.5 posterior cingulate gyrus; 0.9 intracalcarine cortex; 0.6 lingual gyrus
	2872	-61 -11 -15	-5.3	left middle temporal gyrus	L: 82.5 middle temporal gyrus; 13.4 superior temporal gyrus; 3.9 temporal pole
**rmTLE > HC**	7456	-63 -45 30	4.5	left supramarginal gyrus	L: 67.2 supramarginal gyrus; 24.1 angular gyrus; 4.7 lateral occipital cortex; 3.1 superior parietal lobule
	2360	-21 -37 62	4.2	left postcentral gyrus	L: 77.3 postcentral gyrus; 20.3 precentral gyrus; 2.4 superior parietal lobule
**Faces: left anterior hippocampus seed**					
**rmTLE < HC**	5560	24 -19 -17	-5.1	right hippocampus	R: 47.1 hippocampus; 23.2 amygdala; 4.2 pallidum; 5.5 parahippocampal gyrus; 2.0 accumbens; 1.3 putamen
**rmTLE > HC**	2776	30 30 6	4.6	no label	R: 47.0 frontal orbital cortex; 40.6 insular cortex; 5.8 inferior frontal gyrus pars triangularis; 4.6 frontal operculum cortex
**Faces: right anterior hippocampus seed**					
**rmTLE < HC**	7080	-21 -1 -17	-5.5	left amygdala	L: 37.6 hippocampus; 31.2 amygdala; 8.4 temporal fusiform cortex; 9.2 parahippocampal gyrus; 5.9 putamen; 1.8 pallidum; 1.4 frontal orbital cortex; 1.0 temporal occipital fusiform cortex
	5200	2 -53 18	-5.1	right precuneus	L: 41.1 precuneus; 17.4 posterior cingulate gyrus; 3.5 lingual gyrus<br>R: 24.5 precuneus; 12.5 posterior cingulate gyrus
	4072	-9 52 36	-5.4	left frontal pole	L: 53.6 frontal pole; 18.9 superior frontal gyrus<br>R: 20.6 frontal pole; 6.5 superior frontal gyrus
	2864	-1 62 -9	-4.1	right frontal pole	L: 45.5 frontal pole; 11.5 frontal medial cortex;<br>R: 38.0 frontal pole; 3.9 frontal medial cortex

**Words: left anterior hippocampus seed**					
**rmTLE < HC**	5160	18 -13 -19	-5.3	right hippocampus	R: 50.9 hippocampus; 25.7 amygdala; 8.2 parahippocampal gyrus; 2.6 pallidum
**rmTLE > HC**	2448	8 28 40	4.8	right paracingulate gyrus	R: 86.3 paracingulate gyrus; 8.5 anterior cingulate gyrus; 3.6 superior frontal gyrus
**Words: right anterior hippocampus seed**					
**rmTLE < HC**	11184	-7 -59 14	-5.1	left precuneus	L: 43.6 precuneus; 22.8 posterior cingulate gyrus; 1.1 intracalcarine cortex; 1.1 lingual gyrus<br>R: 19.5 precuneus; 10.0 posterior cingulate gyrus; 1.2 lingual gyrus
	5168	-63 -9 -15	-4.8	left middle temporal gyrus	L: 74.1 middle temporal gyrus; 19.0 temporal pole; 5.0 superior temporal gyrus
	4960	-29 -19 -19	-5.0	left hippocampus	L: 56.5 hippocampus; 21.0 amygdala; 11.0 parahippocampal gyrus; 6.8 accumbens; 1.3 subcallosal cortex
	2536	-9 64 22	-3.8	left frontal pole	L: 59.3 frontal pole; 18.9 superior frontal gyrus<br>R: 14.8 superior frontal gyrus; 6.6 frontal pole
	2432	-33 32 -17	-5.2	left frontal orbital cortex	L: 74.0 frontal orbital cortex; 25.0 frontal pole; 1.0 temporal pole
	1776	2 64 -11	-4.2	right frontal pole	L: 38.7 frontal pole; 11.7 frontal medial cortex; 4.5 paracingulate gyrus<br>R: 45.1 frontal pole
	1736	-39 -37 -23	-4.5	left temporal fusiform cortex	L: 80.2 temporal fusiform cortex; 18.0 temporal occipital fusiform cortex
	1488	62 -3 -15	-4.0	left temporal occipital fusiform gyrus	R: 82.3 middle temporal gyrus; 14.0 temporal pole; 3.8 superior temporal gyrus
	16	66 -7 -23	-3.0	right middle temporal gyrus	R: 100 middle temporal gyrus
**rmTLE > HC**	1544	30 48 18	4.8	right frontal pole	R: 99.5 frontal pole

Note. Abbreviations: L, left hemisphere; HC, healthy controls; R, right hemisphere; rmTLE, right mesial temporal lobe epilepsy.

Table S5

*Group comparisons of **A** absolute and **B** relative left and right anterior hippocampus connectivity of lmTLE and FLE patients during scene, face and word encoding.*

	Clustersize [mm^3]	Peak	*t*	Peak Region	Cluster Regions [% of cluster in the respective region]
**A. Group comparison: absolute connectivity: lmTLE patients vs. FLE**					
**Faces: left anterior hippocampus seed**					
**lmTLE < FLE**	4968	28 -31 -13	-5.7	right hippocampus	R: 61.5 hippocampus; 16.3 amygdala; 19.2 parahippocampal gyrus
**lmTLE > FLE**	2184	38 46 24	3.7	right frontal pole	R: 100 frontal pole
	2168	-39 50 6	5.0	left frontal pole	L: 94.1 frontal pole
**Faces: right anterior hippocampus seed**					
**lmTLE < FLE**	2728	-23 -17 -17	-6.1	left hippocampus	L: 79.5 hippocampus; 16.4 amygdala; 1.8 pallidum; 2.1 parahippocampal gyrus
**lmTLE > FLE**	6536	4 -45 22	4.5	right posterior cingulate gyrus	L: 22.5 posterior cingulate gyrus; 15.4 precuneus<br>R: 39.9 posterior cingulate gyrus; 20.0 precuneus
**Words: left anterior hippocampus seed**					
**lmTLE < FLE**	8048	-13 38 56	-5.3	left superior frontal gyrus	L: 65.3 frontal pole; 22.2 superior frontal gyrus; 1.1 paracingulate gyrus<br>R: 5.5 frontal pole; 2.9 superior frontal gyrus; 2.8 paracingulate gyrus
	4448	26 -15 -31	-5.8	right parahippocampal gyrus	R: 55.6 hippocampus; 25.5 parahippocampal gyrus; 15.1 amygdala; 2.9 pallidum
	3800	-43 38 -15	-4.3	left frontal pole	L: 38.1 frontal orbital cortex; 32.6 inferior frontal gyrus pars triangularis; 16.2 frontal pole; 11.0 inferior frontal gyrus pars opercularis
**lmTLE > FLE**	4728	56 -41 56	4.8	no label	R: 51.8 angular gyrus; 43.5 supramarginal gyrus; 4.6 superior parietal lobule
	4200	38 32 34	6.0	right middle frontal gyrus	R: 65.5 frontal pole; 34.5 middle frontal gyrus
**B. Group comparison: relative connectivity: lmTLE patients vs. FLE**					
**Scenes: left anterior hippocampus seed**					
**lmTLE > FLE**	3856	-5 48 40	4.5	left superior frontal gyrus	L: 25.9 superior frontal gyrus; 23.0 frontal pole; 9.8 paracingulate gyrus<br>R: 23.0 frontal pole; 17.4 superior frontal gyrus
**Scenes: right anterior hippocampus seed**					
**lmTLE > FLE**	2736	-7 48 42	4.7	left superior frontal gyrus	L: 39.8 superior frontal gyrus; 33.9 frontal pole<br>R: 14.6 frontal pole; 11.4 superior frontal gyrus
	1944	-35 -75 54	4.5	left lateral occipital cortex	L: 97.9 left lateral occipital cortex
**Words: left anterior hippocampus seed**					
**lmTLE > FLE**	2720	50 -39 46	4.3	right supramarginal gyrus	R: 55.3 angular gyrus; 42.9 supramarginal gyrus
	1656	30 56 6	5.9	right frontal pole	R: 100 frontal pole
	1600	36 34 34	4.4	right middle frontal gyrus	R: 70.0 middle frontal gyrus; 30.0 frontal pole

1480	64 -19 -9	4.4	right middle temporal gyrus	R: 74.6 middle temporal gyrus; 25.4 superior temporal gyrus

Note. Abbreviations: L, left hemisphere; lmTLE, left mesial temporal lobe epilepsy; HC, healthy controls; R, right hemisphere.

Table S6

*Group comparisons of **A** absolute and **B** relative left and right anterior hippocampus connectivity of rmTLE and FLE patients during scene, face and word encoding.*

	Clustersize [mm^3]	Peak	*t*	Peak Region	Cluster Regions [% of cluster in the respective region]
**A. Group comparison: absolute connectivity: rmTLE patients vs. FLE**					
**Scenes: right anterior hippocampus seed**					
**rmTLE < FLE**	2856	-25 -41 -17	-4.5	left temporal fusiform cortex	L: 35.3 parahippocampal gyrus; 27.5 hippocampus; 17.4 temporal fusiform cortex; 13.4 lingual gyrus; 6.2 temporal occipital fusiform cortex
	2608	-7 -57 10	-4.3	left precuneus	L: 69.0 precuneus; 1.8 lingual gyrus; 1.8 posterior cingulate gyrus<br>R: 27.0 precuneus
**rmTLE > FLE**	2752	-59 -49 30	4.7	left supramarginal gyrus	L: 91.6 supramarginal gyrus; 6.4 angular gyrus
	40	-65 -55 38	3.0	no label	L: 20.0 lateral occipital cortex; 20.0 angular gyrus; 20.0 supramarginal gyrus
**Faces: right anterior hippocampus seed**					
**rmTLE < FLE**	4720	-19 -1 -15	-5.9	left amygdala	L: 40.3 hippocampus; 34.9 amygdala; 10.9 putamen; 7.0 pallidum
**Words: left anterior hippocampus seed**					
**rmTLE < FLE**	2544	32 -15 -21	-5.1	right hippocampus	R: 69.2 hippocampus; 20.1 parahippocampal gyrus; 10.7 amygdala
**rmTLE > FLE**	2192	28 56 12	5.4	right frontal pole	R: 100 frontal pole
	1824	32 -67 46	3.3	right lateral occipital cortex	R: 100 lateral occipital cortex
**Words: right anterior hippocampus seed**					
**rmTLE < FLE**	5344	-7 -43 2	-4.8	no label	L: 46.7 precuneus; 21.1 posterior cingulate gyrus; 3.4 lingual gyrus; 3.4 intracalcarine cortex; 2.0 hippocampus; 1.1 supracalcarine cortex<br>R: 16.8 precuneus; 4.2 posterior cingulate gyrus
	2072	-29 -21 -19	-4.9	left hippocampus	L: 65.6 hippocampus; 27.4 parahippocampal gyrus; 7.0 temporal fusiform cortex
	696	-19 -43 -19	-3.7	no label	L: 56.3 parahippocampal gyrus; 11.5 hippocampus; 11.5 temporal fusiform cortex; 6.9 temporal occipital fusiform cortex; 6.9 lingual gyrus
	56	-37 -35 -23	-3.2	left temporal fusiform cortex	L: 100 temporal fusiform cortex
**rmTLE > FLE**	2880	30 50 16	5.8	right frontal pole	R: 93.3 frontal pole; 6.4 middle frontal gyrus
**B. Group comparison: relative connectivity: lmTLE patients vs. FLE**					
**Faces: left anterior hippocampus seed**					
**rmTLE < FLE**	4032	8 64 14	-5.0	right frontal pole	L: 41.7 frontal pole; 11.9 paracingulate gyrus<br>R: 45.2 frontal pole;
**Words: right anterior hippocampus seed**					
**rmTLE < FLE**	5504	-67 -61 -1	-5.6	no label	L: 67.9 middle temporal gyrus; 28.5 lateral occipital cortex; 1.5 inferior temporal gyrus

Note. Abbreviations: L, left hemisphere; HC, healthy controls; R, right hemisphere; rmTLE, right mesial temporal lobe epilepsy.

Table S7

Group comparisons of relative left and right anterior hippocampus connectivity of controls and ***A*** *lmTLE patients* ***B*** *rmTLE patients and* ***C*** *FLE patients during scene, face and word encoding.*

	Clustersize [mm^3]	Peak	*t*	Peak Region	Cluster Regions [% of cluster in the respective region]
**A. Group comparison: relative connectivity: lmTLE patients vs. controls**					
**Scenes: left anterior hippocampus seed**					
**lmTLE < HC**	1664	-33 -67 -15	-4.7	left occipital fusiform gyrus	L: 69.2 occipital fusiform gyrus; 30.8 temporal occipital fusiform cortex
**lmTLE > HC**	2336	-7 62 18	4.1	left frontal pole	L: 51.7 frontal pole; 19.9 paracingulate gyrus; 1.4 paracingulate gyrus<br>R: 16.8 paracingulate gyrus; 9.9 frontal pole
	2208	-5 48 40	5.1	left superior frontal gyrus	L: 51.5 superior frontal gyrus; 33.7 frontal pole<br>R: 11.6 superior frontal gyrus; 3.3 frontal pole
	2096	-47 24 -1	4.0	left frontal operculum cortex	L: 35.1 inferior frontal gyrus pars triangularis; 25.6 frontal orbital cortex; 19.1 frontal operculum cortex; 12.2 inferior frontal gyrus pars opercularis; 7.3 frontal pole
	1952	-39 -59 30	3.8	no label	L: 71.7 lateral occipital cortex; 20.1 angular gyrus; 1.2 supramargial gyrus
	1752	-13 44 8	3.9	left anterior cingulate gyrus	L: 33.3 paracingulate gyrus; 27.4 anterior cingulate gyrus<br>R: 30.6 anterior cingulate gyrus; 8.7 paracingulate gyrus
	56	-15 46 22	3.1	no label	L: 85.7 paracingulate gyrus
**B. Group comparison: relative connectivity: rmTLE patients vs. controls**					
**Scenes: right anterior hippocampus seed**					
**rmTLE < HC**	2928	-33 -63 -11	-4.5	no label	L: 42.4 temporal occipital fusiform cortex; 29.5 occipital fusiform gyrus; 25.7 lingual gyrus
	2008	34 -49 -21	-4.1	Right temporal occipital fusiform cortex	R: 70.5 temporal occipital fusiform cortex; 28.3 occipital fusiform gyrus
	32	44 -47 -11	-3,1	no label	R: 75 inferior temporal gyrus
**rmTLE > HC**	1904	-49 -45 54	4.7	left supramarginal gyrus	L: 76.9 supramarginal gyrus; 15.1 superior parietal lobule; 8.0 angular gyrus
**Faces: right anterior hippocampus seed**					
**rmTLE < HC**	10736	28 -99 -9	-5.4	right occpitial pole	R: 40.5 lateral occipital cortex; 23.4 inferior temporal gyrus; 11.9 occipital fusiform gyrus; 11.8 occipital pole; 5.7 middle temporal gyrus; 5.6 temporal occipital fusiform cortex
	5096	-37 -73 -21	-5.0	left occipital fusiform gyrus	L: 35.3 occipital fusiform gyrus; 22.4 inferior temporal gyrus; 12.4 lateral occipital cortex; 12.1 temporal occipital fusiform cortex; 9.9 occipital pole; 1.7 temporal fusiform cortex
**rmTLE > HC**	1640	-21 28 48	4.1	left superior frontal gyrus	L: 68.3 superior frontal gyrus; 18.5 paracingulate gyrus; 11.7 middle frontal gyrus
	136	-1 42 28	3.4	left paracingulate gyrus	L: 64.7 paracingulate gyrus<br>R: 35.3 paracingulate gyrus
	32	-27 38 38	3.1	left frontal pole	L: 75.0 frontal pole; 25.0 middle frontal gyrus

**C. Group comparison: relative connectivity: FLE patients vs. controls**					
**Scenes: left anterior hippocampus seed**					
**FLE < HC**	4784	28 -65 42	-4.5	right lateral occipital cortex	R: 37.1 supramarginal gyrus; 36.0 lateral occipital cortex; 24.8 angular gyrus; 1.8 superior parietal lobule
	2248	48 30 12	-4.3	Right inferior frontal gyrus pars triangularis	R: 45.9 middle frontal gyrus; 30.6 frontal pole; 23.5 inferior frontal gyrus pars triangularis
	2008	60 -43 12	-4.2	right supramarginal gyrus	R: 45.0 middle temporal gyrus; 44.6 supramarginal gyrus; 10.4 angular gyrus

Note. Abbreviations: FLE, frontal lobe epilepsy; L, left hemisphere; lmTLE, left mesial temporal lobe epilepsy; HC, healthy controls; R, right hemisphere; rmTLE, right mesial temporal lobe epilepsy.
